# Serum metabolites characterize hepatic phenotypes and reveal shared pathways: results from population-based imaging

**DOI:** 10.1186/s10020-025-01309-z

**Published:** 2025-07-21

**Authors:** Juliane Maushagen, Johanna Nattenmüller, Ricarda von Krüchten, Barbara Thorand, Annette Peters, Wolfgang Rathmann, Jerzy Adamski, Christopher L. Schlett, Fabian Bamberg, Rui Wang-Sattler, Susanne Rospleszcz

**Affiliations:** 1 Department of Diagnostic and Interventional Radiology, Medical Center - University of Freiburg, Faculty of Medicine, University of Freiburg, Freiburg, Germany; 2 Institute of Epidemiology, Helmholtz Zentrum München, Neuherberg, Germany; 3 Institute for Radiology and Nuclear Medicine Hirslanden Clinic St. Anna, Lucerne, Switzerland; 4 German Center for Diabetes Research (DZD), Munich-Neuherberg, Germany; 5 Institute for Medical Information Processing, Biometry, and Epidemiology (IBE), Medical Faculty, Ludwig-Maximilians-Universität (LMU), Munich, Germany; 6 German Center for Cardiovascular Disease Research (DZHK), Munich Heart Alliance, Munich, Germany; 7 Institute for Biometrics and Epidemiology, German Diabetes Center (DDZ), Leibniz Center for Diabetes Research at Heinrich Heine University Düsseldorf, Düsseldorf, Germany; 8 Institute of Experimental Genetics, Helmholtz Zentrum München, Neuherberg, Germany; 9 Department of Biochemistry, Yong Loo Lin School of Medicine, National University of Singapore, Singapore, Singapore; 10 Institute of Biochemistry, Faculty of Medicine, University of Ljubljana, Ljubljana, Slovenia; 11 Institute of Translational Genomics, Helmholtz Zentrum München, Neuherberg, Germany

**Keywords:** Steatotic liver disease, NAFLD, Liver fat, Liver iron, Metabolomics

## Abstract

**Background:**

Steatotic liver disease is a major public health issue, with hepatic iron overload exacerbating fibrotic conditions. This study aimed to identify metabolites associated with hepatic fat and/or iron overload using targeted metabolomics in a population-based cohort.

**Methods:**

We used the cross-sectional KORA-MRI study (*N* = 376 individuals). Hepatic fat and iron content were derived by magnetic resonance imaging, and serum metabolite concentrations were quantified through targeted metabolomics. Associations between 146 metabolites and 40 indicators with hepatic phenotypes were analyzed, adjusted for confounders, and corrected for multiple testing. Formal pathway analyses and mediation analyses including genetic data were conducted. Performance of metabolomics to diagnose steatosis or hepatic iron overload was evaluated using ROC curves, and compared to the fatty liver index (FLI).

**Results:**

Overall, 50.8% of participants (mean age 56.4 years) had hepatic steatosis, and 43.6% iron overload. Twelve unique metabolites/indicators (amino acids, lysophosphatidylcholine, acyl-alkyl-phosphatidylcholine), and sums of branched chain and aromatic amino acids, and five lipids, and ratio of acyl-alkyl-phosphatidylcholines to diacyl-phosphatidylcholines were associated with hepatic fat content. 27 metabolites/indicators, including 25 lipids, were associated with hepatic iron content. Addition of these metabolites to the FLI improved diagnosis of steatosis and iron overload nominally. Glycerophospholipid metabolism, phenylalanine, tyrosine and tryptophan biosynthesis and glycerophospholipid metabolism were shared pathway between steatosis and iron overload. Alanine, isoleucine, glutamine and pimeloylcarnitine (C7-DC) mediated effects between genetic variants and hepatic phenotypes.

**Conclusion:**

Metabolites were associated with hepatic fat and iron content, shared common pathways, and improved diagnosis of steatosis and iron overload, highlighting the role of iron in hepatic disorders.

**Supplementary Information:**

The online version contains supplementary material available at 10.1186/s10020-025-01309-z.

## Background

Hepatic steatosis is one of the most common chronic liver diseases and a major public health concern. The sharply increased prevalence of steatotic liver disease (SLD) (encompassing metabolic dysfunction-associated steatotic liver disease (MASLD); formerly non-alcoholic fatty liver disease, NAFLD) has been recently estimated as 23.4% worldwide (Paik et al., 2023). This is mainly due to the parallel increase in obesity and diabetes that are major risk factors. However, also individuals without metabolic impairment are at risk of developing hepatic steatosis. Particularly these individuals are difficult to identify as a high-risk group, which prevents timely treatment and fosters disease progression.

Recently, genetic variants in the *HFE* gene, predisposing to hepatic hemochromatosis, were found to be associated with hepatic steatosis in individuals with regular body mass index (BMI) (Sun et al. 2023). Moreover, hepatic iron has been implicated to exacerbate fibrotic conditions in recent animal studies (Altamura et al. 2021). Excess iron has lipotoxic effects, is known to induce inflammation and promote oxidative stress (Altamura et al. 2021) and is implied in the pathogenesis of insulin resistance (Harrison et al. 2023). A comprehensive analysis corroborated that patterns of iron deposition in hepatocytes and reticuloendothelial cells are associated with histologic features and steatosis severity (Nelson et al. 2011). Since progression of hepatic steatosis to fibrosis and cirrhosis is a main cause of hepatic death, pathways implied in iron metabolism could be relevant targets for therapeutic interventions. Increased circulating iron has been suggested to play a causal role in risk for hepatic steatosis (Sun et al. 2024).

Metabolomics has emerged as a powerful tool to characterize pathophysiological pathways in metabolic disease. Specific serum metabolites have been shown to be associated with distinct stages of liver disease (McGlinchey et al. 2022). Other metabolites have been reported to distinguish between different subtypes of MASLD (Shao et al., 2023) and an atlas of metabolites for hepatic triglyceride content is mainly characterized by two groups of metabolites (Faquih et al. 2023). Moreover, associations between iron metabolism markers with the plasma lipidome and metabolome have been reported from population-based studies (Kaul et., 2018).

Given the substantial public health burden of SLD, it is necessary to investigate the role of hepatic iron in pathways to liver disease on a population-based level, including individuals without overt hepatic disease. Further metabolomics-based characterization of underlying metabolic pathways between genetics, hepatic iron, and SLD will improve our understanding of pathophysiology of SLD, might identify metabolites that can be used for diagnosis or prediction of phenotypes, and identify potential treatment targets, e.g. with respect to iron-related pathways. Current non-invasive markers to determine risk for SLD are limited in their ability to identify intermediate disease stages (Wong et al. 2018), and current recommended treatment for SLD in Europe is solely lifestyle based without medication regimes. However, as stated above, not all individuals with steatosis present overt metabolic risk factors that lend themselves to lifestyle interventions.

In the current study, we aimed to use population-based data to identify serum metabolites that are associated with hepatic phenotypes, i.e. hepatic fat and iron content derived by magnetic resonance imaging (MRI), and liver enzymes. We hypothesized that there are distinct serum metabolites associated with these phenotypes that provide a better characterization of metabolic pathways from genetic variants to hepatic outcomes and diagnostic of hepatic steatosis and iron overload.

## Methods

### Study sample

We use data from the KORA-MRI study, which is a sub-study of the prospective, population-based KORA-S4 cohort (Cooperative Health Research in the Region of Augsburg, *N* = 4261, enrolled in 1999/2001) in Southern Germany. The data of the current analysis are cross-sectional and include *N* = 400 participants from the second follow-up in 2013/2014 (KORA-FF4, *N* = 2279). While the KORA-S4 cohort is drawn from the general population in the region of Augsburg, the KORA-MRI is a subsample focused on cardiovascular risk across the glycemic spectrum in individuals free of cardiovascular diseases (CVD). The framework of the KORA-MRI study has been described previously (Bamberg et al. 2017). Individuals were eligible for enrollment if they were younger than 74 years, had no cardiovascular disease (myocardial infarction, stroke, revascularization), and no contraindications to MRI, including adequate renal function (serum creatinine < 1.3 mg/dL). Whole-body MRI at a central imaging site took place within three months after a detailed examination at the KORA study center, including a face-to-face interview with trained interviewers, a physical examination, and a blood draw after an overnight fast.

All KORA cohorts are approved by the Ethics Committee of the Bavarian Association of Physicians (EC# 06068). The MRI study was additionally approved by the institutional review board of the Medical Faculty of the Ludwig-Maximilians-University Munich (# 498–12). The study was conducted according to the Declaration of Helsinki, and all participants provided written informed consent.

### Outcome: hepatic phenotypes

Hepatic fat and iron content were measured on a high-speed T2-corrected multi-echo sequence on 3 T MRI (Magnetom Skyra, Siemens Healthineers, Erlangen, Germany) in the left and right liver lobe. Hepatic fat content was assessed in % and iron content as relaxation rate in 1/s. Continuous values were averaged over the left and right liver lobe. Hepatic steatosis was defined as fat content ≥ 5.56% and iron overload as values ≥ 41.0 s^−1 (^Kühn et al. 2017; Szczepaniak et al. 2005). Since the study was population-based, no liver biopsy or histology were done.

Liver enzymes gamma-glutamyl transferase (GGT), alanine transaminase (ALT) and aspartate transaminase (AST) were measured in fasted serum samples in μkat/l by the modified IFCC method. Additionally, the fatty liver index (FLI) was calculated from BMI, waist circumference, triglycerides and GGT according to the published formula (Bedogni et al. 2006). A FLI < 30 is suggested to rule out prevalent SLD and a FLI > 60 is suggested to diagnose prevalent SLD (Bedogni et al. 2006). We chose the FLI as a reference score, because it is an established tool that is readily available and applicable in clinical practice to estimate potential steatosis, especially in the absence of histological examination.

### Exposure: targeted metabolomics

Targeted serum metabolites were quantified from fasted samples by the AbsoluteIDQ™ p180 kit (BIOCRATES Life Sciences AG, Innsbruck, Austria). The advantage of a targeted approach over untargeted metabolomics is the high precision in identification and (relative) quantification of metabolites. Especially the high precision allows for better comparability among different studies, particularly when no validation cohort is accessible. For the AbsoluteIDQ™ p180 kit interlaboratory reproducibility showed high precision (Siskos et al. 2017) and the application in various cohort studies (Floegel et al., 2013; Kaul et., 2018) supports reliability of the targeted metabolomics approach. The kit measures different biochemical groups of amino acids, biogenic amines, carnitines, lysophosphatidylcholines (lysoPC), sphingomyelins (SM), diacylphosphatidylcholines (diacylPC), acylalkylphosphatidylcholines (acylalkylPC) and hexoses.

For sample preparation, 10 µl of serum was added to the 96-well kit plate, along with the respective internal standards for LC separation to the filter inserts, which already contained internal standards for the FIA analysis. Derivatization of amino acids and biogenic amines was done with 5% phenylisothiocyanate in ethanol/water/pyridine. After extraction of metabolites and internal standards with methanol/5 mM ammonium acetate solvent, acetonitrile/water and the kits running solvent were added for LC–MS/MS (C18 guard column) and FIA-MS/MS analysis, respectively. Samples were randomly distributed across 29 plates, including five pooled EDTA-plasma reference samples from Sera Laboratories International Ltd. (Hull, United Kingdom) (Zukunft et al. 2018). MetIQ™ software was used for metabolite identification (Dong et al., 2023) quantifying metabolites in µmol/l. During quality control, those metabolites were removed where the coefficient of variance was ≥ 25% in reference sampels, or the limit of detection per plate was ≥ 50% of all metabolite concentrations, or the non-detectable rate was ≥ 50%, resulting in a final number of 146 metabolites (Maushagen et al. 2024). Additionally, 40 metabolite indicators were calculated out of the 146 metabolite levels as ratios and sums of metabolites to capture effects of chemical groups or as proxies for enzyme activity (Supplementary Table 1). Subsequent data processing was stratified by sex: Data were winsorized to the 95% percentile, logarithmized and then standardized (minus mean and divided by standard deviation) by plate to account for potential batch effects.

### Genotyping

Genotyping was done with the Affymetrix Axiom Chip and subsequent imputation with HRC panel 1.1 (Maier et al., 2021). We selected rs738409 (reference|effect allele C|G) in *PNPLA3* as the lead SNP for hepatic fat content (Trépo et al. 2016) and rs1800562 (reference|effect allele G|A), in *HFE* as the lead single nucleotide polymorphism (SNP) for hepatic iron content (Wilman et al. 2019).

### Clinical covariates

Smoking behavior, menopausal status and medication intake were self-reported. Daily alcohol consumption was calculated from self-reported type of beverages consumed and consumption frequency. Hypertension was defined as systolic blood pressure ≥ 140 mmHg and/or diastolic blood pressure ≥ 90 mmHg, or intake of antihypertensive medication while being aware of having hypertension. Lipid profile was measured in fasted serum samples by enzymatic, colorimetric Flex assays (Vista, Siemens or Cobas, Roche). In addition to HbA1c, fasting glucose and fasting insulin, individuals without a diagnosis of type 2 diabetes additionally underwent an oral glucose tolerance test and were categorized as normoglycemia, prediabetes, or type 2 diabetes based the results according to World Health Organization criteria.

### Statistical analyses

Participant characteristics are given as means and standard deviation for continuous data and counts and percentages for categorical data. The relation between hepatic fat and hepatic iron parameters were described using Spearman’s correlation coefficients. Principal components analysis of metabolomics data was done to assess data quality. Associations of metabolite exposures with hepatic phenotypes were evaluated by linear or logistic regression models. For linear models, continuous hepatic outcomes (hepatic fat and iron content, enzymes) were logarithmized before modeling and resulting estimates denote percent change in geometric mean with corresponding 95% confidence intervals (CI). For logistic models (steatosis and iron overload), odds ratios with corresponding 95% CI are given. Models were calculated for the whole sample and sex-stratified. First, regressions were adjusted for age (years), sex (if not sex-stratified) and BMI (kg/m^2^). Second, full adjustment included additionally glycemia status (normoglycemia, prediabetes, diabetes), systolic blood pressure (mmHg), serum triglycerides (mg/dL) and daily alcohol consumption (g/d). All *p*-values were corrected for False Discovery Rate (FDR) using the Benjamini–Hochberg method and values < 0.05 were considered statistically significant.

To understand the relationship between genes, metabolites and hepatic phenotypes we performed formal mediation analysis using a regression-based approach. Associations of post-imputation dosages of lead SNPs with 1) the hepatic phenotypes fat and iron content and, 2) metabolites identified as significantly associated with hepatic fat or steatosis, or iron content or iron overload, were assessed by linear regression with confounder adjustment as above on the whole sample. For metabolites significantly associated with SNPs, formal mediation analysis was performed, using the R package “*mediation*”, for the pathway SNP- > metabolite- > hepatic phenotype. The “*mediation*” package uses the framework introduced by Imai et al. and decomposes the average total effect into a direct and mediated effect (Imai et al. 2010).

As a first step to assess the ability of metabolites to diagnose, or improve the diagnosis of, the categorical parameters hepatic steatosis and iron overload, logistic regressions were calculated using leave-one-out cross validation. We refrained from statistical prediction of continuous parameters, since the precise prediction of subclinical amounts of hepatic fat and iron is clinically less relevant compared to prediction of pathologic phenotypes. However, metabolites from all outcomes (hepatic fat/steatosis or hepatic iron/iron overload) were used to capture as many metabolites as possible on the pathway to disease. We computed the corresponding receiver operating characteristic (ROC) curves with Area under the curve (AUC) as a performance measure. Three models were calculated with the predictors 1) FLI alone, 2) metabolites/indicators that were significantly associated with the respective phenotype, 3) FLI plus metabolites/indicators from model 2) for the respective phenotype. As an additional analysis, we explored whether hepatic iron and post-imputation dosages of lead SNPs predict hepatic steatosis, and whether hepatic fat content and post-imputation dosages of lead SNPs predict hepatic iron overload without additional variables.

R version 4.1.1 was used for all calculations.

### Pathway analysis

Two pathway analyses (PAs) were conducted to identify different pathways between individuals with and without steatosis, and individuals with and without iron overload, respectively. The PAs were done with MetaboAnalyst 5.0 (Pang et al., 2021), which includes 80 possible pathways for homo sapiens from the Kyoto Encyclopedia of Genes and Genomes database. The analysis combines an enrichment analysis of metabolite sets using Fisher’s exact test with a topology analysis using relative-betweenness centrality. Pathways with an FDR corrected *p*-value < 0.05 were considered significantly enriched. Metabolites significantly associated with any hepatic outcome were used for the PAs.

## Results

### Characterization of study sample

One participant retroactively withdrew their consent for data usage. Participants with incomplete data on hepatic MRI or metabolites were excluded (Supplementary Fig. 1), resulting in a sample size of 217 men and 159 women. Genetic data were available for 351 participants.

Individuals with hepatic steatosis or iron overload were significantly older, had higher body weight, higher alcohol consumption, and higher diabetes prevalence compared to individuals without (Table 1). Hepatic fat content and hepatic iron content were significantly higher in individuals with iron overload and hepatic steatosis, respectively (Table 1). Participants were, on average, 56 years old, with two-thirds of the women being post-menopause (Supplementary Table 2). Almost two-thirds of men and one third of women had hepatic steatosis as defined by MRI, with an average hepatic fat content of 10.8% in men and 6.2% in women (Supplementary Table 2). Among men with a FLI > 60, which is suggested to identify steatotic liver disease, 81.1% had steatosis as defined by MRI, while among women with a FLI > 60, 70.4% had steatosis as defined by MRI. Additionally, 55.3% of men and 27.7% of women had iron overload (Supplementary Table 2).

**Table 1:** Characteristics of the total sample and stratified by hepatic steatosis and iron overload

	**Overall**	Stratified according to hepatic steatosis			Stratified according to hepatic iron overload		
		**Steatosis present**	**Steatosis absent**	***p*-value**	**Iron overload present**	**Iron overload absent**	***p*-value**
**n**	**376**	**191**	**185**		**164**	**212**	
Age, years	56.4 (9.2)	58.2 (8.6)	54.6 (9.4)	< 0.001	58.3 (8.5)	55.0 (9.4)	< 0.001
Sex, male		139 (72.8)	78 (42.2)	< 0.001	120 (73.2)	97 (45.8)	< 0.001
Weight, kg	82.9 (16.7)	91.0 (15.6)	74.6 (13.3)	< 0.001	87.3 (14.8)	79.5 (17.3)	< 0.001
Height, cm	171.8 (9.8)	173.2 (9.9)	170.3 (9.5)	0.004	173.7 (9.9)	170.3 (9.4)	0.001
BMI, kg/m2	28.0 (4.9)	30.3 (4.6)	25.7 (4.0)	< 0.001	28.9 (4.3)	27.4 (5.2)	0.002
Waist circumference, cm	98.4 (14.4)	106.8 (12.2)	89.8 (11.0)	< 0.001	102.8 (12.3)	95.1 (15.0)	< 0.001
Post menopause	106 (66.7)	44 (23.0)	62 (33.5)	< 0.001	39 (23.8)	67 (31.6)	< 0.001
Alcohol consumption, g/day (median [IQR])	8.6 [0.2, 26.2]	16.94 [34.3]	5.71 [20.0]	< 0.001	19.6 [34.92]	4.4 [19.5]	< 0.001
Smoking behaviour				< 0.001			0.086
Never	137 (36.4)	66 (34.6)	71 (38.4)		82 (50.0)	82 (38.7)	
Former	164 (43.6)	102 (53.4)	62 (33.5)		54 (32.9)	83 (39.2)	
Current	75 (19.9)	23 (12.0)	52 (28.1)		28 (17.1)	47 (22.2)	
Systolic blood pressure, mmHg	120.6 (16.9)	126.7 (16.7)	114.3 (14.6)	< 0.001	124.9 (16.9)	117.3 (16.1)	< 0.001
Diastolic blood pressure, mmHg	75.3 (10.0)	78.6 (10.0)	72.0 (8.9)	< 0.001	77.4 (9.3)	73.7 (10.3)	< 0.001
Hypertension	129 (34.3)	34 (18.4)	95 (49.7)	< 0.001	72 (43.9)	57 (26.9)	0.001
Antihypertensive medication	97 (25.8)	68 (35.6)	29 (15.7)	< 0.001	52 (31.7)	45 (21.2)	0.029
Total cholesterol, mg/dL	217.6 (36.6)	218.3 (37.5)	216.9 (35.6)	0.712	219.2 (36.8)	216.5 (36.4)	0.479
Triglycerides, mg/dL	131.7 (86.7)	159.4 (90.8)	103.2 (72.1)	< 0.001	148.4 (93.9)	118.9 (78.6)	0.001
LDL, mg/dL	139.4 (33.1)	141.4 (33.6)	137.2 (32.5)	0.223	140.4 (33.7)	138.5 (32.7)	0.586
Lipid lowering medication	41 (10.9)	32 (16.8)	9 (4.9)	< 0.001	21 (12.8)	20 (9.4)	0.383
Glycemia				< 0.001			0.001
Normoglycemic	232 (61.7)	81 (42.4)	151 (81.6)		84 (51.2)	148 (69.8)	
Prediabetes	91 (24.2)	68 (35.6)	23 (12.4)		52 (31.7)	39 (18.4)	
T2 Diabetes	53 (14.1)	42 (22.0)	11 (5.9)		28 (17.1)	25 (11.8)	
HbA1c, %	5.6 (0.7)	5.7 (0.7)	5.5 (0.7)		5.6 (0.6)	5.6 (0.8)	0.557
Fasting glucose, mg/dL	104.1 (22.9)	110.5 (24.1)	97.6 (19.5)	< 0.001	107.2 (21.4)	101.7 (23.7)	0.02
Fasting insulin, mg/dL	11.2 (7.7)	14.9 (8.7)	7.5 (3.7)	< 0.001	12.9 (8.1)	10.0 (7.1)	< 0.001
Serum uric acid, mg/dL	5.6 (1.5)	6.3 (1.4)	4.9 (1.3)	< 0.001	6.2 (1.5)	5.2 (1.4)	< 0.001
hsCRP, mg/L (median [Q1, Q3])	1.18 [0.61, 2.47]	1.56 [2.3]	0.84 [1.5]	< 0.001	1.2 [2.2]	1.1 [1.8]	0.194
**Hepatic phenotypes**							
Hepatic fat content, %	8.9 (8.1)	14.50 (7.98)	3.04 (1.29)	< 0.001	12.30 (8.95)	6.20 (6.25)	< 0.001
Hepatic fat content, %, median [IQR]	5.7 [9.1]	11.8 [12.3]	2.9 [2.0]		9.4 [14.5]	4.0 [5.5]	
Steatosis	191 (50.8)				114 (69.5)	77 (36.3)	< 0.001
Hepatic iron content, 1/s	40.6 (4.7)	42.43 (4.42)	38.74 (4.27)	< 0.001	44.75 (2.88)	37.41 (3.09)	< 0.001
Iron overload	164 (43.6)	114 (59.7)	50 (27.0)	< 0.001			
ALT (GPT), μkat/l	0.52 (0.29)	0.48 (0.61)	0.40 (0.23)	< 0.001	0.58 (0.31)	0.47 (0.27)	0.001
AST (GOT), μkat/l	0.42 (0.22)	0.46 (0.16)	0.38 (0.25)	< 0.001	0.44 (0.15)	0.41 (0.25)	0.085
GGT, μkat/l	0.66 (0.67)	0.83 (0.68)	0.48 (0.61)	< 0.001	0.79 (0.72)	0.56 (0.61)	0.001
Fatty liver index, continuous	54.2 (31.3)	73.51 (22.53)	34.18 (26.17)	< 0.001	64.82 (27.19)	45.91 (31.87)	< 0.001
< 30	109 (29.0)	9 (4.7)	100 (54.1)		22 (13.4)	87 (41.0)	
≥ 30 and < 60	86 (22.9)	41 (21.5)	45 (24.3)		37 (22.6)	49 (23.1)	
≥ 60	181 (48.1)	141 (73.8)	40 (21.6)		105 (64.0)	76 (35.8)	

Presented are mean (SD) for continuous data or n (%) for categorical data if not stated differently. Hepatic steatosis was defined as hepatic fat content ≥ 5.56%. Iron overload was defined as hepatic iron content ≥ 41 1/s

In sex-stratified correlation analyses log-transformed hepatic fat and hepatic iron showed low to moderate positive correlations (female: r = 0.53, *p* < 0.001; male: r = 0.31, *p* < 0.001; Supplementary Fig. 2). The PCA plot showed no outliers for metabolomic data and did not reveal any batch effects, indicating valid data quality (Supplementary Fig. 3).

### Association of serum metabolites with hepatic phenotypes

Adjusting for age, sex and BMI showed significant associations between 45 metabolites/3 indicators and hepatic fat content and 16 metabolites with steatosis of which 13 metabolites were overlapping between hepatic fat content and steatosis. Moreover, 26 metabolites were associated with iron content and 28 metabolites/9 indicators with hepatic iron overload of which 20 metabolites overlapped between iron and iron overload (Additional File 1).

**Fig. 1:**
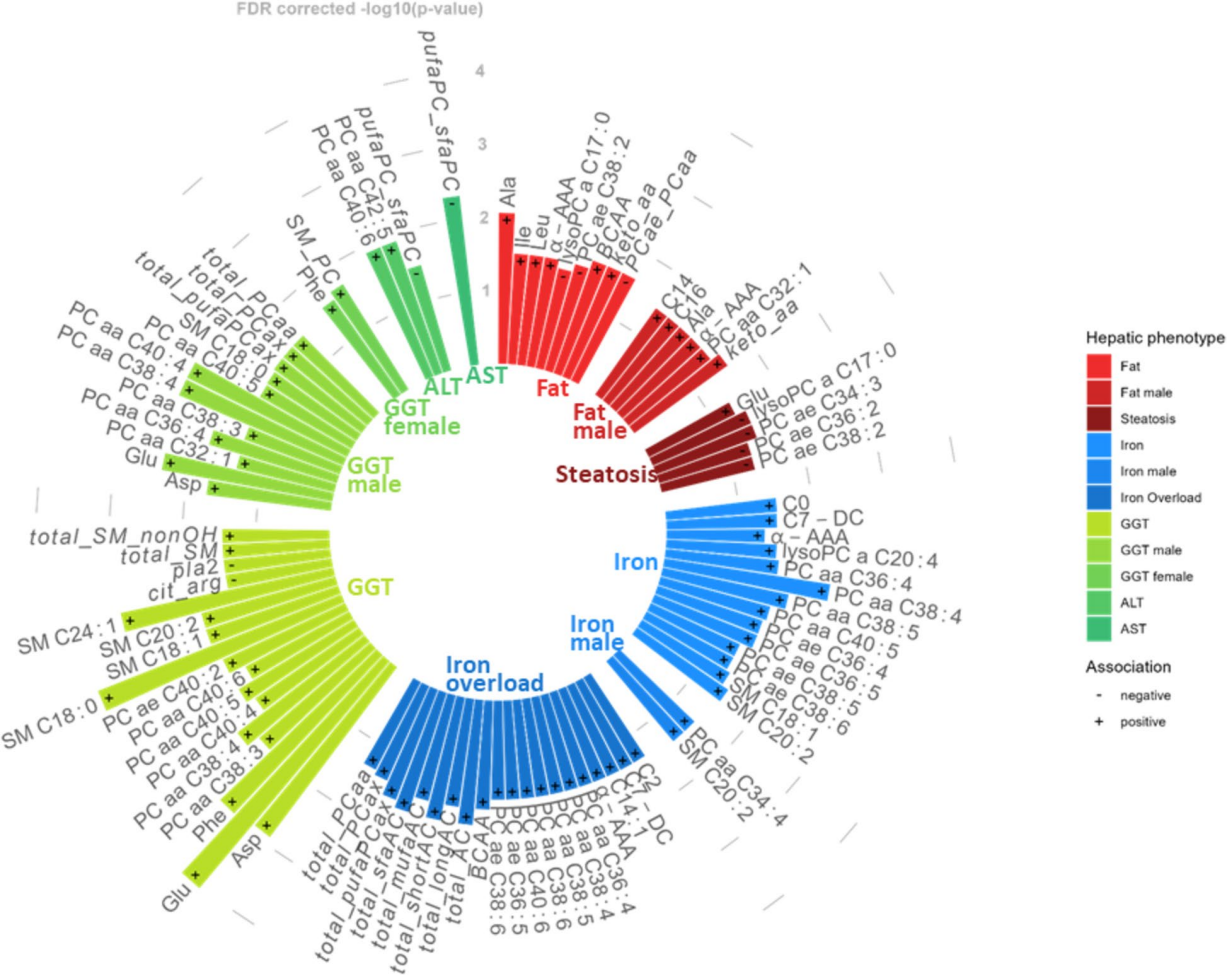
Circular bar plot of all significant associations between metabolites, metabolite indicators (name printed in italics) and hepatic phenotypes, for the whole sample and sex-stratified. Colors of bars indicate hepatic phenotype. Positive associations are marked with “ + ” and negative associations with “-”. Bar heights correspond to -log10 (FDR corrected p-value). GGT = gamma-glutamyl transferase, ALT = alanine transaminase (GPT), AST = aspartate transaminase (GOT)

After full adjustment 9 metabolites/indicators (Ala, Ile, Leu, alpha-AAA, lysoPC a C17:0, PC ae C38:2, bcaa, keta_aa, PCae_PCaa) were significantly associated with hepatic fat content, and 5 metabolites (Glu, lysoPC a C17:0, PC ae C34.3, PC ae C36:2, PC ae C38:2) were associated with steatosis. Two metabolites (lysoPC a C17:0, PC ae C38:2) overlapped between these phenotypes. In total, 12 unique metabolites/indicators significantly associated with hepatic fat content or steatosis (Fig. 1, Supplementary Fig. 4, Table 2, Additional File 2).

**Table 2:** Significant associations of metabolites/indicators with the respective hepatic phenotype adjusted for the full model

Metabolite or Indicator	Estimate (95% CI)	FDR corrected *p*-value	Log_10_ p-value
Fat			
Ala	1.17 (1.08, 1.26)	0.009	2.06
Ile	1.13 (1.05, 1.22)	0.031	1.51
Leu	1.13 (1.05, 1.22)	0.031	1.51
alpha.AAA	1.14 (1.05, 1.23)	0.031	1.51
lysoPC a C17.0	0.88 (0.81, 0.95)	0.04	1.39
PC ae C38.2	0.88 (0.82, 0.95)	0.031	1.51
bcaa	1.13 (1.05, 1.21)	0.024	1.62
keto_aa	1.13 (1.05, 1.21)	0.024	1.62
PCae_PCaa	0.86 (0.79, 0.94)	0.024	1.62
Fat male			
C14	1.19 (1.08, 1.31)	0.037	1.44
C16	1.18 (1.07, 1.3)	0.037	1.44
Ala	1.19 (1.07, 1.31)	0.037	1.44
alpha.AAA	1.18 (1.07, 1.31)	0.037	1.44
PC aa C32.1	1.21 (1.08, 1.35)	0.037	1.44
keto_aa	1.18 (1.07, 1.3)	0.027	1.57
Hepatic steatosis			
Glu	1.72 (1.24, 2.41)	0.049	1.31
lysoPC a C17.0	0.55 (0.39, 0.76)	0.035	1.46
PC ae C34.3	0.56 (0.4, 0.76)	0.035	1.46
PC ae C36.2	0.59 (0.42, 0.81)	0.049	1.31
PC ae C38.2	0.62 (0.45, 0.83)	0.049	1.31
Iron			
C0	1.02 (1.01, 1.03)	0.031	1.52
C7.DC	1.02 (1.01, 1.03)	0.031	1.51
Alpha.AAA	1.02 (1.01, 1.03)	0.046	1.34
Lysopc a C20.4	1.02 (1.01, 1.03)	0.031	1.51
PC aa C36.4	1.02 (1.01, 1.04)	0.028	1.55
PC aa C38.4	1.03 (1.01, 1.04)	0.005	2.29
PC aa C38.5	1.02 (1.01, 1.04)	0.018	1.75
PC aa C40.5	1.02 (1.01, 1.04)	0.028	1.55
PC ae C36.4	1.02 (1.01, 1.03)	0.031	1.51
PC ae C36.5	1.02 (1.01, 1.03)	0.031	1.51
PC ae C38.5	1.02 (1.01, 1.03)	0.046	1.34
PC ae C38.6	1.02 (1.01, 1.03)	0.046	1.34
SM C18.1	1.02 (1.01, 1.03)	0.04	1.4
SM C20.2	1.02 (1.01, 1.03)	0.031	1.51
Iron male			
PC aa C34.4	1.04 (1.02, 1.05)	0.038	1.42
SM C20.2	1.03 (1.01, 1.05)	0.038	1.42
Iron overload			
C2	1.53 (1.18, 1.98)	0.045	1.35
C7.DC	1.46 (1.14, 1.88)	0.045	1.35
C14.1	1.49 (1.16, 1.93)	0.045	1.35
Alpha.AAA	1.5 (1.15, 1.96)	0.045	1.35
PC aa C36.4	1.53 (1.18, 2.01)	0.045	1.35
PC aa C38.4	1.5 (1.15, 1.97)	0.045	1.35
PC aa C38.5	1.52 (1.17, 1.99)	0.045	1.35
PC aa C38.6	1.57 (1.21, 2.05)	0.045	1.35
PC aa C40.6	1.51 (1.17, 1.97)	0.045	1.35
PC ae C36.5	1.46 (1.14, 1.89)	0.045	1.35
PC ae C38.6	1.45 (1.14, 1.87)	0.045	1.35
Bcaa	1.45 (1.12, 1.88)	0.033	1.49
Total_AC	1.52 (1.18, 1.97)	0.02	1.71
Total_longAC	1.44 (1.12, 1.86)	0.033	1.49
Total_shortAC	1.56 (1.21, 2.02)	0.02	1.71
Total_mufaAC	1.43 (1.11, 1.86)	0.033	1.49
Total_sfaAC	1.52 (1.18, 1.98)	0.02	1.71
Total_pufaPCax	1.5 (1.16, 1.96)	0.025	1.61
Total_PCax	1.44 (1.1, 1.88)	0.039	1.41
Total_PCaa	1.43 (1.09, 1.88)	0.045	1.34
GGT			
Asp	1.14 (1.07, 1.21)	0.001	2.89
Glu	1.19 (1.11, 1.27)	0	4.04
Phe	1.15 (1.08, 1.22)	0.001	2.96
PC aa C38.3	1.13 (1.06, 1.22)	0.01	2
PC aa C38.4	1.13 (1.06, 1.21)	0.007	2.19
PC aa C40.4	1.11 (1.04, 1.19)	0.019	1.71
PC aa C40.5	1.12 (1.05, 1.19)	0.015	1.83
PC aa C40.6	1.11 (1.04, 1.18)	0.025	1.6
PC ae C40.2	1.11 (1.04, 1.18)	0.015	1.83
SM C18.0	1.16 (1.09, 1.23)	0	3.57
SM C18.1	1.12 (1.05, 1.19)	0.013	1.9
SM C20.2	1.11 (1.05, 1.18)	0.013	1.89
SM C24.1	1.14 (1.07, 1.21)	0.001	2.96
Cit_arg	0.91 (0.85, 0.97)	0.035	1.46
Pla2	0.9 (0.84, 0.97)	0.035	1.46
Total_SM	1.1 (1.03, 1.17)	0.035	1.46
total_SM_nonOH	1.1 (1.03, 1.17)	0.035	1.46
GGT male			
Asp	1.14 (1.06, 1.23)	0.02	1.71
Glu	1.19 (1.09, 1.29)	0.004	2.36
PC aa C32.1	1.15 (1.05, 1.27)	0.043	1.37
PC aa C36.4	1.16 (1.07, 1.27)	0.015	1.82
PC aa C38.3	1.15 (1.05, 1.26)	0.042	1.38
PC aa C38.4	1.19 (1.1, 1.29)	0.004	2.43
PC aa C40.4	1.2 (1.1, 1.3)	0.004	2.43
PC aa C40.5	1.14 (1.05, 1.24)	0.037	1.43
SM C18.0	1.13 (1.05, 1.22)	0.036	1.45
Total_pufaPCax	1.14 (1.05, 1.23)	0.033	1.48
Total_PCax	1.15 (1.05, 1.25)	0.033	1.48
Total_PCaa	1.15 (1.05, 1.25)	0.033	1.48
GGT female			
Phe	1.23 (1.1, 1.36)	0.026	1.58
SM_PC	1.23 (1.09, 1.37)	0.02	1.69
ALT			
PC aa C40.6	1.09 (1.04, 1.14)	0.012	1.91
PC aa C42.5	1.09 (1.04, 1.14)	0.012	1.91
pufaPC_sfaPC	0.92 (0.88, 0.97)	0.032	1.49
AST			
pufaPC_sfaPC	0.93 (0.89, 0.96)	0.005	2.32

*Abbreviations CI* Confidence interval

For hepatic iron content, 14 metabolites (C0, C7-DC, alpha-AAA, lysoPC a C20:4, PC aa C36:4, PC aa C38:4, PC aa C38:5, PC aa C40:5, PC ae C36:4, PC ae C36:5, PC ae C38:5, PC ae C38:6, SM C18:1, SM C20:2), but no indicators, were significantly associated after adjustment. Additionally, 20 metabolites/indicators (C2, C7-DC, C14:1, alpha-AAA, PC aa C36:4, PC aa C38:4, PC aa C38:5, PC aa C38:6, PC aa C40:6, PC ae C36:5, PC ae C38:6, bcaa, total_AC, total_longAC, total_shortAC, total_mufaAC, total_sfaAC, total_pufaPCax, total_PCax, total_PCaa) were significantly associated with hepatic iron overload, with 7 overlapping metabolites (C7-DC, alpha-AAA, PC aa C36:4, PC aa C38:4, PC aa C38:5, PC ae C36:5, PC ae C38:6) between these two phenotypes. In total, 27 metabolites/indicators were associated with hepatic iron content or hepatic iron overload (Fig. 1, Supplementary Fig. 4, Table 2, Additional File 2).

Additionally, 17 metabolites/indicators were associated with GGT, AST, and ALT. Overall, 38 unique metabolites and 17 unique indicators were identified to be associated with at least one hepatic phenotype (Fig. 1, Supplementary Fig. 4, Table 2, Additional File 2).

While there were no sex-specific associations of any metabolite with the outcomes of steatosis or iron overload 5 metabolites and 1 indicator were associated with hepatic fat content in men only, and 2 metabolites were associated with hepatic iron in men only (Additional File 2). There was no overlap between men and women in metabolites or indicators associated with GGT (Fig. 1, Supplementary Fig. 4, Additional File 2).

### Role of serum metabolites in the pathway between genetic variants and hepatic phenotypes

**Fig. 2:**
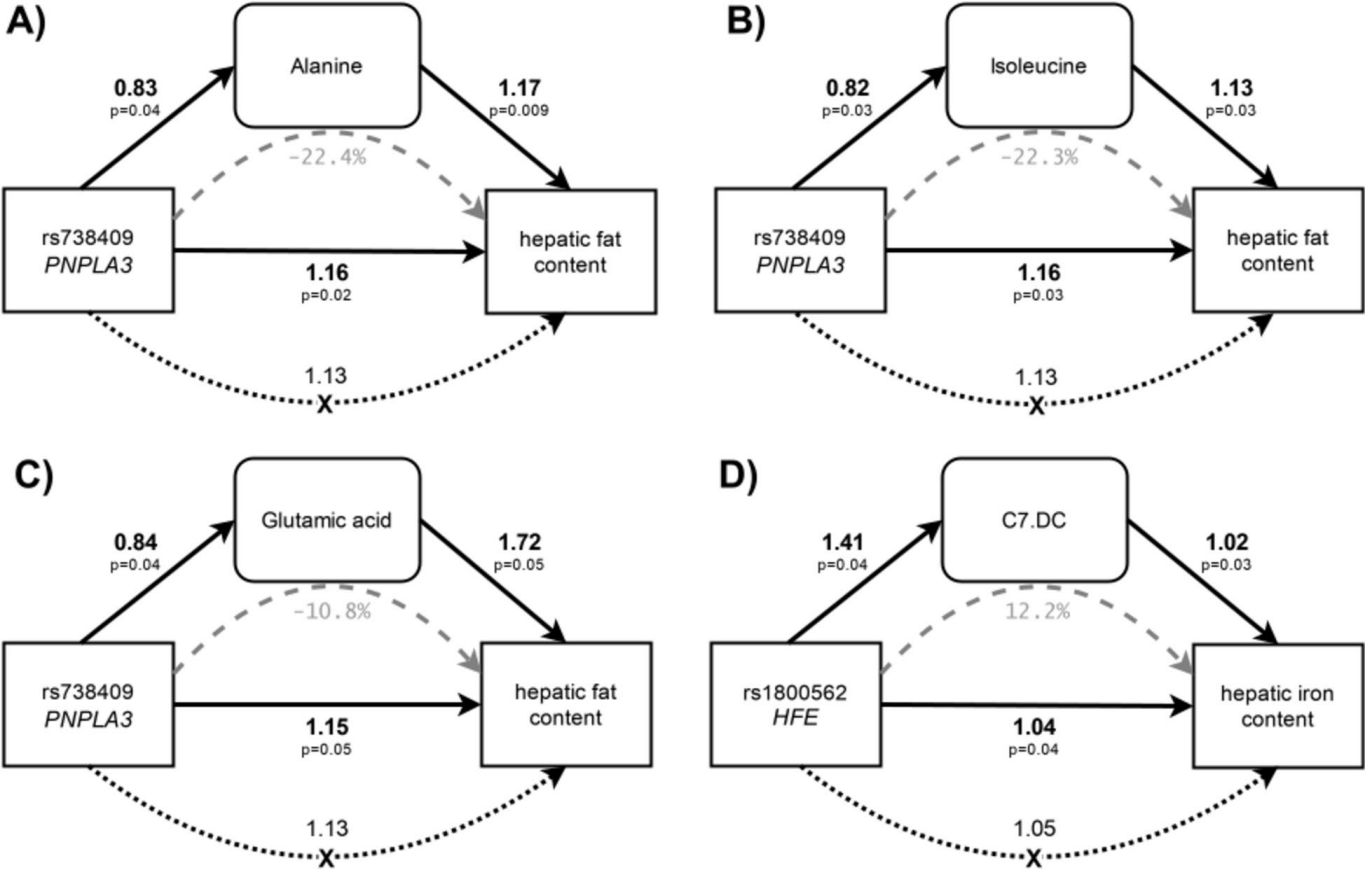
Mediation analysis (**A**-**C**) show associations of rs738409 (PNPLA3) with hepatic fat content and **D**) shows associations of rs1800562 (HFE) with hepatic iron content. Solid arrows indicate direct effects; dashed lines indicate the mediated proportion, and the dotted lines show the total effect via unknown (X) pathways. Note that the total effect for (**A-C**) is smaller than the direct effect, due to the suppression through the mediators (corresponds to negative proportion mediated)

After full adjustment, rs738409 (*PNPLA3*) was tentatively associated with hepatic fat (estimate = 1.13, 95%CI: [0.99; 1.29], *p* = 0.07), but not iron content (estimate = 1.00, 95%CI 0.98; 1.02], *p* = 0.92, Table 3), whereas rs1800562 (*HFE*) was tentatively associated with increased fat (estimate = 1.3, 95%CI [0.99; 1.59], *p* = 0.06), and iron content (estimate = 1.05, 95%CI [1.01; 1.09], *p* = 0.02, Table 3). After full adjustment, rs738409 (*PNPLA3*) was negatively associated with the amino acids alanine, isoleucine and glutamate (Table 3) and rs1800562 (*HFE*) was positively associated with acylcarnitine C7DC (Table 3). Formal mediation analysis showed that alanine, isoleucine and glutamate were suppressors of the association between rs738409 and hepatic fat (Fig. 2A-C), attenuating the direct effect of the SNP. In comparison, 12.2% of the association between rs1800562 and hepatic iron were mediated by acylcarnitine C7DC (Fig. 2D).

**Table 3:** Association of SNPs with hepatic phenotypes and metabolites

		**Unadjusted**		**Base adjustment**		**Full adjustment**	
SNP (*Gene*)	Outcome	Estimate [95% CI]	*p*-value	Estimate [95% CI]	*p*-value	Estimate [95% CI]	*p*-value
rs738409 (*PNPLA3*)	Hepatic fat	1.19 [0.99; 1.43]	0.06	1.13 [0.98; 1.30]	0.10	1.13 [0.99; 1.29]	0.07
	Hepatic iron	1.01 [0.98; 1.03]	0.53	1.00 [0.98; 1.03]	0.84	1.00 [0.98; 1.03]	0.92
	Alanine	0.83 [0.69; 0.99]	0.04	0.82 [0.68; 0.99]	0.04	0.83 [0.04; 0.99]	0.04
	Isoleucine	0.81 [0.68; 0.98]	0.03	0.81 [0.67; 0.97]	0.02	0.82 [0.69; 0.98]	0.03
	Glu	0.83 [0.69; 1.0]	0.05	0.82 [0.68; 0.99]	0.04	0.84 [0.71; 0.99]	0.04
rs1800562 (*HFE*)	Hepatic fat	1.51 [1.09; 2.10]	0.01	1.33 [1.03; 1.72]	0.03	1.25 [0.99; 1.59]	0.06
	Hepatic iron	1.06 [1.02; 1.11]	0.01	1.05 [1.01; 1.10]	0.01	1.05 [1.01; 1.09]	0.02
	C7.DC	1.44 [1.03; 2.02]	0.03	1.42 [1.02; 1.97]	0.04	1.41 [1.01; 1.96]	0.04

The base model is adjusted for age (years), sex and BMI (kg/m^2^) and the full adjustment additionally for glycemia status (normoglycemia, prediabetes, diabetes), systolic blood pressure (mmHg), serum triglycerides (mg/dL) and daily alcohol consumption (g/d)

### Difference in pathways

**Fig. 3:**
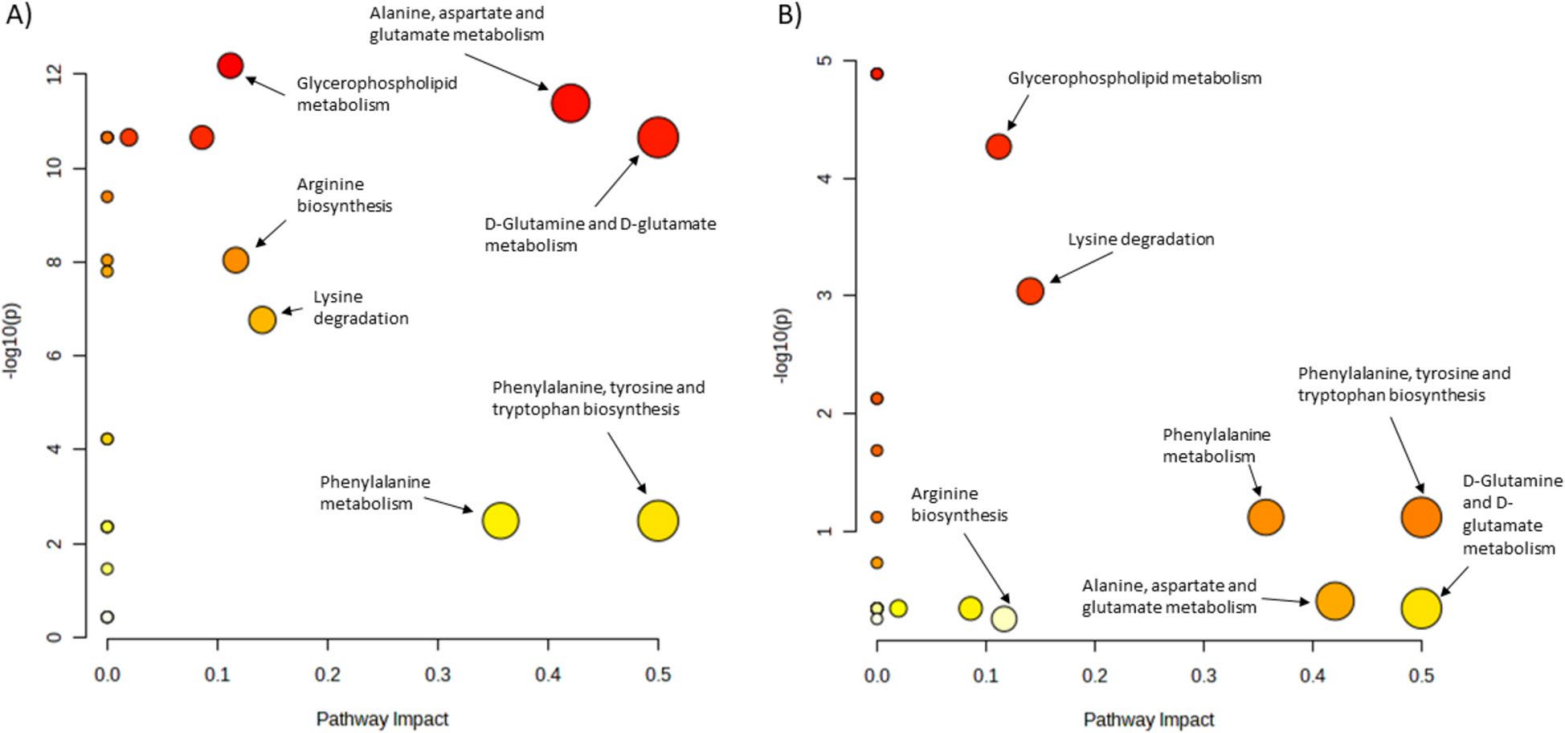
Pathway analyses in individuals with steatosis and iron overload (**A**) Comparison of pathways in individuals with and without steatosis. **B** Comparison of pathways in individuals with and without iron overload. Colors indicate enriched pathways displayed as -log10 (*p*-value). Yellow = lower enrichment, red = higher enrichment. Circle size indicates pathway impact. Please note the different scaling of y-axes

Using any significant metabolite from the regression analysis in the pathway analysis, individuals with steatosis or iron overload showed several different enriched pathways, with effects on other pathways (Fig. 3, Supplementary Table 3–4). In individuals with steatosis, the d-glutamine and d-glutamate metabolism or alanine, aspartate and glutamate metabolism showed strong impacts. The glycerophospholipid metabolism, arginine biosynthesis and lysine degradation were significantly enriched in the steatosis group. In individuals with iron overload the same four pathways showed a high topology impact, however, enrichment was low. Furthermore, glycerophospholipid metabolism and lysine degradation were enriched (Fig. 3, Supplementary Table 3–4).

### Discrimination of hepatic steatosis and iron overload

**Fig. 4:**
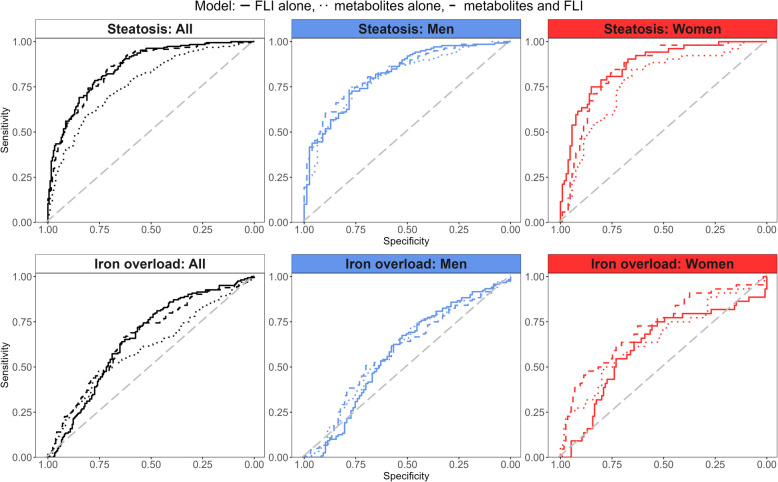
Discrimination of steatotic liver disease and hepatic iron overload. Upper panel: ROC curves for outcome steatosis with predictors FLI (solid line), 12 metabolites/indicators (dotted line) and FLI plus Ala, Ile, Leu, alpha-AAA, lysoPC a C17:0, PC ae C38:2, Glu, PC ae C34:3, PC ae C36:2, BCAA, keto_aa, PCae_PCaa (dashed line). Lower panel: ROC curves for outcome iron overload with predictors FLI (solid line), 28 metabolites/indicators (dotted line) and FLI plus C0, C2, C7-DC, C14:1, alpha-AAA, BCAA, lysoPC a C20:4, PC aa C36:4, PC aa C38:4, PC aa C38:5, PC aa C38:6, PC aa C40:5, PC aa C40:6, PC ae C36:4, PC ae C36:5, PC ae C38:5, PC ae C38:6, SM C18:1, SM C20:2, total AC, total longAC, total shortAC, total mufaAC, total sfaAC, total pufaPCax, total PCax, total PCaa (dashed line)

For discrimination of hepatic steatosis, the continuous FLI showed an AUC of 0.859 in the overall sample (Table 4, Fig. 4, upper panel). The 12 metabolites/indicators significantly associated with hepatic fat content or hepatic steatosis showed a significantly lower AUC of 0.769 (*p *< 0.001). Adding the 12 metabolites/indicators to the FLI slightly decreased discrimination nominally (AUC of 0.851), but not significantly (*p* = 0.336, Table 4, Fig. 4).

**Table 4:** Areas under the Curve (AUC) from leave-one-out cross validation logistic regression models predicting hepatic phenotypes

Outcome: Group	Predictor	AUC	*p*-value
Steatosis: All	FLI alone	0.859	1
	Metabolites alone	0.769	< 0.001
	Metabolites and FLI	0.851	0.3335
Steatosis: Men	FLI alone	0.818	1
	Metabolites alone	0.802	0.6362
	Metabolites and FLI	0.83	0.5935
Steatosis: Women	FLI alone	0.867	1
	Metabolites alone	0.771	0.0189
	Metabolites and FLI	0.844	0.1439
Iron overload: All	FLI alone	0.658	1
	Metabolites alone	0.602	0.1328
	Metabolites and FLI	0.664	0.8414
Iron overload: Men	FLI alone	0.579	1
	Metabolites alone	0.589	0.8288
	Metabolites and FLI	0.587	0.8387
Iron overload: Women	FLI alone	0.613	1
	Metabolites alone	0.657	0.5375
	Metabolites and FLI	0.723	0.0722

Values are given for different groups (all, men, women) based on combinations of fatty liver index (FLI) and metabolites. *P*-values are retrieved from comparing to the model including FLI alone

In men, the continuous FLI showed an AUC of 0.818. The 12 metabolites/indicators showed nominally (AUC of 0.802), but not significantly (*p* = 0.636), worse discrimination of hepatic steatosis. Adding the 12 metabolites/indicators to the FLI improved discrimination nominally (AUC of 0.830), but not significantly *p* = 0.594, Table 4, Fig. 4). In women, the continuous FLI showed an AUC of 0.867. The 12 metabolites/indicators showed a significantly worse discrimination (AUC of 0.771, *p* = 0.019). Adding the 12 metabolites/indicators to the FLI declined discrimination nominally (AUC of 0.844), but again not significantly (*p* = 0.144, Table 4, Fig. 4).

While hepatic steatosis was better discriminated by lead SNPs for *HFE* gene compared to hepatic iron content, the lead SNP for *PNPLA3* gene showed smaller AUCs than hepatic iron content in additional analyses (Supplementary Table 5).

The discrimination of hepatic iron overload was not statistically significant in any model. For discrimination of hepatic iron overload, the continuous FLI showed an AUC of 0.658 in the overall sample (Table 4, Fig. 4, lower panel). The 27 metabolites/indicators significantly associated with hepatic iron content or hepatic iron overload showed a nominally lower AUC of 0.602 (*p* = 0.180). Adding the 27 metabolites/indicators to the FLI improved discrimination nominally (AUC of 0.664, *p* = 0.841, Table 4, Fig. 4).

In men, the continuous FLI showed an AUC of 0.579. The 27 metabolites/indicators showed a nominally better discrimination of hepatic iron overload (AUC of 0.589, *p* = 0.829). Adding the 27 metabolites/indicators to the FLI improved discrimination nominally compared to FLI alone, but was slightly worse than metabolites alone (AUC of 0.587, *p* = 839). In women, the continuous FLI showed an AUC of 0.613. The 27 metabolites/indicators alone and in combination with the FLI (AUC of 0.657 and 0.723, respectively) improved discrimination nominally (*p* = 0.538 and *p* = 0.072, respectively, Table 4, Fig. 4). Generally, for the discrimination of iron overload there was a consistent improvement from FLI alone, over metabolites/indicators alone to the combination of both in sex-specific analyses.

## Discussion

Our analysis of the association of serum metabolites with hepatic phenotypes resulted in 91 associations of 38 unique metabolites and 23 associations of 17 unique metabolite indicators. Pathway analyses showed differences in amino acid related pathways between individuals with and without steatosis, and the same pathways exhibited a high impact in iron overload, indicating an overlap between pathways of steatosis and iron overload on a molecular level. We identified mediating roles of metabolites in the association of genetic variants with hepatic phenotypes, and showed that metabolites have the potential to improve the diagnostic performance of the established FLI.

It has already been shown that circulating metabolites are associated with stages of hepatic steatosis in a patient cohort, illustrating the potential of these measurements to reflect the process of fatty liver pathogenesis, and to serve as early biomarkers of hepatic deterioration (McGlinchey et al. 2022). In a recent publication of the population-based Rotterdam Study, with three replication samples, the association of metabolites with SLD revealed several lipids and amino acids to be involved in SLD pathology (Abozaid et al. 2025). Especially our results of amino acids coincide with these findings, while lipids partly show other effect directions. We identified overlapping pathways in steatosis and iron overload such as amino acid metabolism and glycerophospholipid metabolism. These pathways were found before in the progression from liver cirrhosis to hepatocellular carcinoma (Gao et al. 2015). We hypothesize that these pathways and their metabolites might describe early disease progression from non-severe steatosis to MASLD, underlining the role of iron in this process.

### Amino acids

We found the sum of branched-chain amino acids (BCAA) to be associated with both liver fat and iron overload, and amino-acid related pathways were enriched or had significant impact in pathway analysis (Fig. 3A and Supplementary file 3). BCAA levels in humans are maintained by a complex interplay of BCAA digestion and catabolism, and increased BCAAs levels are associated with several diseases including insulin resistance and diabetes. One well-studied effect of leucine is the protein synthesis or cell growth via the mechanistic target of rapamycin (mTOR) pathway (Neinast et al. 2019).

A systematic review showed that circulating BCAAs were associated with NAFLD in the majority of studies (Piras et al., 2021). Kalhan et al. (2011) showed increased BCAAs and glutamate levels in individuals with non-alcoholic steatohepatitis (NASH) compared to healthy individuals, but no associations of BCAAs with steatosis (Kalhan et al. 2011). Männistö et al. (Männistö et al. 2015) found lower concentrations of BCAAs in individuals with steatosis compared to NASH patients. This indicates that BCAA concentrations increase with hepatic disease severity, supporting our findings that BCAA were associated with hepatic fat content in early disease stages. A cross-sectional analysis of a randomized controlled trial confirms a gradual increase of circulating BCAA with increase in hepatic fat content (Amanatidou AI et al. 2023).

Direction and causality of the association between BCAA and adipose tissue increase are still unclear. Animal studies suggest that the effect of BCAA also depends on nutrition background (Newgard 2012; Solon-Biet et al. 2022); for instance, insulin resistance is induced by high fat diet in combination with BCAA (Newgard et al. 2009), due to a downregulation of BCAA catabolism in obesity (White et al. 2018). Downstream effects of BCAA are mediated through mTOR complex 1 (mTORC1) which increases oxidative stress in mitochondrial pathways, promotes inflammation via NF-kB or increases hepatic fat content via lipogenesis (Zhenyukh et al. 2017). The same pathway is hypothesized to be involved in iron homeostasis (Guan and Wang 2014), however, iron is also involved in the activation of mTORC1 (Shapiro et al. 2023). Correlations of BCAAs with circulating iron markers have been reported (Enko et al. 2020), and values of these BCAAs were lower in patients with anemia. Moreover, dietary intake of BCAAs have been found to be associated with increased ferritin and hepatic iron content (Galarregui et al. 2021), supporting our results. Another study suggests that iron overload induces liver damage via the inhibiting PI3K/AKT/mTOR signaling pathway in chicks (Lv et al. 2024).

We thus hypothesize that hepatic fat and iron content both contribute to SLD disease progression via BCAA and their downstream effects probably mainly via mTORC1. However, to confirm and assess this pathway, future studies should assess the effect of hepatic fat and iron on SLD progression via BCAA in longitudinal studies or include interventions to evaluate the effect of lowering BCAA.

### Alpha aminoadipic adic

In our analysis, alpha aminoadipic acid (alpha-AAA) was found to be significantly associated with hepatic fat content, iron content and iron overload, and the association with steatosis pointed to the same direction (FDR corrected *p*-value = 0.08). Alpha-AAA in humans is mainly generated by the oxidation of lysine through the saccharopine pathway, and is known to induce a pro-oxidative milieu and generate oxidative stress through impaired mitochondrial function (Estaras et al. 2020). The latter is associated with metabolic syndrome (Prasun 2020) and alpha-AAA levels predict T2D risk (Razquin et al. 2019). Changes in alpha-AAA are associated with adipogenesis; expression is found in adipocytes, but not in preadipocytes, and levels of alpha-AAA are higher in individuals with obesity (Lee et al. 2019), where levels were also correlated with higher cholesterol and glucose. Desine et al. replicated the association of alpha-AAA levels with unfavorable lipid profile and hyperinsulinemia in individuals with HIV. Moreover, they could also show that the association with obesity is indeed driven by adipose tissue dysfunction, since they obtained associations with visceral and hepatic fat, but not subcutaneous fat or anthropometric markers of body size (Desine et al. 2023). Previous analyses on our sample showed a strong association of glycemic traits with both hepatic iron and fat (Niedermayer et al. 2023), as well as an association of visceral adipose tissue with hepatic iron (Maier et al. 2021). In a cell study, alpha-AAA was shown as marker of oxidation of proteins in the presence of iron and under pathological glucose conditions (Luna et al. 2021). We might thus hypothesize that the association of alpha-AAA with hepatic phenotypes is modulated by impaired glycemia. To link the hepatic phenotypes of our analysis we further hypothesize, that elevated levels of alpha-AAA might result of oxidative stress due to iron overload and that alpha-AAA can in turn increase hepatic fat via adipogenesis and therefore promote hepatic steatosis progression. However, the relation of hepatic iron and metabolomics, and in particular alpha-AAA, is currently understudied in population-based cohorts. We therefore suggest that longitudinal studies should assess the relation and direction of associations between hepatic iron, alpha-AAA and hepatic steatosis to further characterize their relationship.

### Lipids

In the current analysis, glycerophospholipids, sphingomyelins and their indicators showed 56 associations with hepatic phenotypes, and several metabolites were associated with more than one phenotype. Moreover, glycerophospholipid metabolism was identified as one of the main pathways differing in individuals with and without steatosis or iron overload. Several of the metabolites identified have been reported before in the context of excess weight and obesity, again indicating the close connection between general metabolic and hepatic impairment. We replicated the association of lysoPC a C17:0 with hepatic fat and steatosis, that was previously implied in other studies, either directly (Feldman et al. 2017), or indirectly through risk factors associated (Prada et al. 2021). In an analysis of three population-based studies, higher levels of lysoPC a 17:0 were protective against abdominal weight gain in both men and women (Merz et al. 2016) and several diacyl and acyl-alkyl PCs, among them PC ae C38:2 and PC ae C36:2 were reported as protective against abdominal weight gain in women. In line with these findings, our current results showed that higher levels of serum phospholipids lysoPC a 17:0 and the acylalkyl-PC ae 38:2 were associated with lower hepatic fat content. Elevated serum lipid levels correspond to lower uptake into hepatocytes, a process driven by phospholipase A_2 (_Stremmel et al. 2014). We thus hypothesize that higher serum PC levels might indicate lower hepatic fat.

LysoPC a C20:4, in our study associated with hepatic iron, has been shown to be associated with circulating ferritin in both men and women (Kaul et al. 2018). Iron overload is associated with hepatic ferroptosis (Chen et al. 2023), a cell death which depends on lipid peroxidation and leads to liver fibrosis. LysoPCs are recycled to PCs by the enzyme LPCAT, which is also a key enzyme in ferroptosis (Pandrangi et al. 2022). Ferroptosis requires mainly PCs esterified with polyunsaturated fatty acids (PUFA), which might explain the associations of iron overload with PUFA lipids as single metabolites and the sum of PUFA PCs in our analysis. PC aa 40:5, as another lipid containing PUFA, is associated with increased risk for diabetes (Floegel et al. 2013) and hepatic fat content (Boone et al. 2019). In our analysis, differently saturated diacyl-PCs of the fatty acid C40 were associated with iron parameters and GGT. We can thus hypothesize that lipids esterified with PUFA, such as lysoPC a C20:4 or PC aa 40:5, are markers of both iron metabolism and metabolic impairment, indicating a shared molecular background.

In our study, especially lipid metabolites (PCs and SMs), that were associated with GGT had overlap with metabolites associated with hepatic steatosis and iron parameters, indicating a connection between pathophysiologic pathways. Indeed, GGT is a marker oxidative stress as it provides reduced glutathione to reduce oxygen species (Irie et al. 2012). Oxidative stress is one important driver of SLD via inflammation (Altamura et al. 2021) and can be induced by hepatic iron.

### Mediation of genetic effects

Effects of rs738409 (*PNPLA3*) on hepatic fat and rs1800562 (*HFE*) on hepatic iron are established (Sun et al. 2023), and were replicated in our sample. The subsequent formal mediation analysis indicated that 12.2% of the effect of rs1800562 on hepatic iron content was mediated through acylcarnitine C7-DC, suggesting a potentially causal, but in any case intermediary role. In line with this finding, acylcarnitines have been reported to be associated with iron hemoglobin and ferritin (Kaul et al. 2018). Interestingly, we found that rs738409 (*PNPLA3*) decreases serum levels of alanine, isoleucine and glutamate (Fig. 2A-C), which in turn are associated with higher hepatic fat content. The association of another variant in *PNPLA3* (rs738408-C) with decreased glutamate has been found before, supporting our results (Richardson et al. 2022). Consequently, the mediation analysis suggests that the pathway between rs738409 and hepatic fat content is partly suppressed through the association with these three amino acids (Fig. 2A-C).

Here, we have assessed only two lead SNPs, as a first step to evaluate the mediating role of metabolites in the pathway between genetic predisposition and hepatic phenotypes. However, to fully investigate these pathways, a more comprehensive panel of genetic variants will be needed, including SNPs in *TM6SF2*, *MBOAT7*, *GCKR*, or *APOE*, which should be analyzed in further studies.

### Sex specific effects and predictions

Previously, we could show that the associations between hepatic iron and risk factors vary according to sex (Maier et al. 2021; Niedermayer et al. 2023). In the current study, we failed to identify consistent sex-specific associations of individual metabolites with hepatic phenotypes, which is likely due to the limited sample size in sex-stratified analyses. Variation in hepatic fat and iron content was lower in women compared to men, and prevalence of steatosis and iron overload was also lower in women, thus the likelihood to identify effects in women was smaller. In line with this, adding metabolites to the FLI improved prediction performance of hepatic steatosis nominally, but not significantly, only in men. The FLI was not designed to identify hepatic iron overload; however, in our sample its ability to distinguish between individuals with and without hepatic iron overload was 0.658 and nominally improved by adding metabolites associated with hepatic iron. We must stress that this model has to be regarded as a very first step, only indicating the potential of these metabolites to improve diagnosis, since due to lack of an external validation sample, we tested diagnostic performance on the same data as the metabolites were derived. We refrained from testing other non-invasive liver scores designed for phenotypes of fibrosis or cirrhosis, such as FIB-4, FNI, NFS, or MASEF. Since biopsy data or elastography imaging was not available in our study to define fibrotic or cirrhotic phenotypes, we cannot properly evaluate the performance of these scores. However, the need to improve the performance of conveniently accessible scores such as the FLI have been recognized (Jung et al. 2020). Metabolite data can be measured from a simple blood draw, and due to improvements in speed, reliability, and cost of metabolite measurements, they have the potential to be easily applicable in clinical practice. Should the predictive performance of the metabolites identified in the current study prove to be robust by validation in further analyses, they would have the potential to somewhat reduce the currently very large grey area of the FLI in diagnosing hepatic steatosis and iron overload.

### Strength and limitations

A main strength of our analysis is the well-characterized study sample with accurate, MRI-based measurements of hepatic fat and iron content, a broad panel of targeted metabolite data as well as ample clinical data for appropriate confounder adjustment. Nevertheless, our analysis has certain limitations. Due to the population-based setting of our study, no biopsies were conducted and we cannot establish presence of fibrosis or cirrhosis. Furthermore, no standard cut off values for iron overload based on MRI exist. We therefore rely on cutoff values, which were calibrated with histopathologic measurements in other cohorts (Kühn et al. 2017). Since our data were cross-sectional, we could not assess causality or the direction of associations and cannot establish if serum metabolites have an impact on hepatic phenotypes, or if metabolites are a consequence of hepatic phenotypes. Additionally, metabolite levels are potentially influenced by a large number of risk factors, including medication, alcohol consumption, and nutrition, so residual confounding remains in our analyses. The sample size prevented us from conducting more detailed, sex-specific analyses with accounting for menopause status. Therefore, studies with longitudinal settings are required to evaluate predictive performance and direction of association.

## Conclusions

In conclusion, findings from our population-based data show that circulating serum metabolites can reflect hepatic phenotypes and are involved in shared pathways, such as lysine degradation and glycerophospholipid metabolism. Iron-related pathways in hepatic steatosis might therefore be an interesting target for intervention.

## Supplementary Information

Additional file 1.

Additional file 2.

Additional file 3.

## Data Availability

Data are available upon request by means of a project agreement from KORA. Requests should be sent to kora.passt@helmholtz-munich.de and are subject to approval by the KORA Board. Analysis codes are available from the authors upon reasonable request.

## Abbreviations

acylalkylPC: Acylalkylphosphatidylcholines
alpha-AAA: Alpha aminoadipic acid
ALT: Alanine transaminase
AST: Aspartate transaminase
AUC: Area under the curve
BCAA: Branched-chain amino acids
BMI: Body mass index
CI: Confidence interval
CVD: Cardiovascular diseases
diacylPC: Diacylphosphatidylcholines
FDR: False discovery rate
FLI: Fatty liver index
GGT: Gamma-glutamyl transferase
KORA: Cooperative Health Research in the Region of Augsburg
lysoPC: Lysophosphatidylcholines
MASLD: Metabolic dysfunction-associated steatotic liver disease
MRI: Magnetic resonance imaging
mTOR: Mechanistic target of rapamycin
mTORC1: MTOR complex 1
NAFLD: Non-alcoholic fatty liver disease
NASH: Non-alcoholic steatohepatitis
PA: Pathway analysis
PUFA: Polyunsaturated fatty acids
ROC: Receiver operating curve
SLD: Steatotic liver disease
SM: Sphingomyelins
SNP: Single nucleotide polymorphism
