# Supplementary material for: Serum metabolites characterize hepatic phenotypes and reveal shared pathways: results from population-based imaging
Source: Mol Med. 2025 Jul 21;31:260. doi: 10.1186/s10020-025-01309-z (PMC12282023; doi:10.1186/s10020-025-01309-z)

Supplementary Material to

**Serum metabolites characterize hepatic phenotypes and reveal shared pathways: results from population-based imaging**

Juliane Maushagen, Johanna Nattenmüller, Ricarda von Krüchten, Barbara Thorand, Annette Peters, Wolfgang Rathmann, Jerzy Adamski, Christopher L. Schlett, Fabian Bamberg, Rui Wang-Sattler, Susanne Rospleszcz

**Supplementary Table 1:** Metabolite indicators: Sums and ratios

| **Metabolite indicator** | **Short name** | **Formula** |
| --- | --- | --- |
| Fischer Ratio | fischer_ratio | (Ile + Leu + Val) / (Phe + Trp + Tyr) |
| Global Arginine Bioavailability Ratio | GABR | Arg/ (Orn + Cit) |
| Ratio of Proline to Citrulline | pro_cit | Pro / Cit |
| Sum of Aromatic Amino Acids | aaa | Phe + Trp + Tyr |
| Sum of Branched-Chain Amino Acids | bcaa | Ile + Leu + Val |
| Sum of Solely Glucogenic Amino Acids | gluco_aa | Ala + Arg + Asn + Asp + Gln + Glu + Gly + His + Met + Pro + Ser + Thr + Val |
| Sum of Solely Ketogenic Amino Acids | keto_aa | Leu + Lys |
| Asymmetrical Arginine Methylation | adma_arg | ADMA / Arg |
| Methionine Oxidation | met_ox | Met.SO / Met |
| Nitric Oxide-Synthase Activity | cit_arg | Cit / Arg |
| Ornithine Synthesis | orn_arg | Orn / Arg |
| Beta-Oxidation | beta_ox | (C2 + C3) / C0 |
| Sum of Acylcarnitines | total_AC | C2 + C3 + C3.DC..C4.OH. + C4 + C4.1 + C5 + C7.DC + C8 + C9 + C10 + C10.1 + C12 + C12.1 + C14 + C14.1 + C14.1.OH + C14.2 + C14.2.OH + C16 + C16.1.OH + C16.2 + C18 + C18.1 + C18.2 |
| Sum of Long-Chain Acylcarnitines | total_longAC | C14 + C14.1 + C14.1.OH + C14.2 + C14.2.OH + C16 + C16.1.OH + C16.2 + C18 + C18.1 + C18.2 |
| Sum of Medium-Chain Acylcarnitines | total_medAC | C7.DC + C8 + C9 + C10 + C10.1 + C12 + C12.1 |
| Sum of Monounsaturated Fatty Acid Acylcarnitines | total_mufaAC | C4.1 + C10.1 + C12.1 + C14.1 + C14.1.OH + C16.1.OH + C18.1 |
| Sum of Polyunsaturated Fatty Acid Acylcarnitines | total_pufaAC | C14.2 + C14.2.OH + C16.2 + C18.2 |
| Sum of Saturated Fatty Acid Acylcarnitines | total_sfaAC | C2 + C3 + C3.DC..C4.OH. + C4 + C5 + C7.DC + C8 + C9 + C10 + C12 + C14 + C16 + C18 |
| Sum of Short-Chain Acylcarnitines | total_shortAC | C2 + C3 + C3.DC..C4.OH. + C4 + C4.1 + C5 |
| Omega-Oxidation | omega_ox | (C3.DC..C4.OH. + C7.DC) / (C2 + C3 + C3.DC..C4.OH. + C4 + C4.1 + C5 + C7.DC + C8 + C9 + C10 + C10.1 + C12 + C12.1 + C14 + C14.1 + C14.1.OH + C14.2 + C14.2.OH + C16 + C16.1.OH + C16.2 + C18 + C18.1 + C18.2) |
| Phospholipase A2 Activity | pla2 | (lysoPC.a.C16.0 + lysoPC.a.C16.1 + lysoPC.a.C17.0 + lysoPC.a.C18.0 + lysoPC.a.C18.1 + lysoPC.a.C18.2 + lysoPC.a.C20.3 + lysoPC.a.C20.4) / (PC.aa.C28.1 + PC.aa.C30.0 + PC.aa.C32.0 + PC.aa.C32.1 + PC.aa.C32.2 + PC.aa.C32.3 + PC.aa.C34.1 + PC.aa.C34.2 + PC.aa.C34.3 + PC.aa.C34.4 + PC.aa.C36.1 + PC.aa.C36.2 + PC.aa.C36.3 + PC.aa.C36.4 + PC.aa.C36.5 + PC.aa.C36.6 + PC.aa.C38.0 + PC.aa.C38.1 + PC.aa.C38.3 + PC.aa.C38.4 + PC.aa.C38.5 + PC.aa.C38.6 + PC.aa.C40.2 + PC.aa.C40.3 + PC.aa.C40.4 + PC.aa.C40.5 + PC.aa.C40.6 + PC.aa.C42.0 + PC.aa.C42.1 + PC.aa.C42.2 + PC.aa.C42.4 + PC.aa.C42.5 + PC.aa.C42.6 + PC.ae.C30.0 + PC.ae.C32.1 + PC.ae.C32.2 + PC.ae.C34.0 + PC.ae.C34.1 + PC.ae.C34.2 + PC.ae.C34.3 + PC.ae.C36.0 + PC.ae.C36.1 + PC.ae.C36.2 + PC.ae.C36.3 + PC.ae.C36.4 + PC.ae.C36.5 + PC.ae.C38.0 + PC.ae.C38.1 + PC.ae.C38.2 + PC.ae.C38.3 + PC.ae.C38.4 + PC.ae.C38.5 + PC.ae.C38.6 + PC.ae.C40.1 + PC.ae.C40.2 + PC.ae.C40.3 + PC.ae.C40.4 + PC.ae.C40.5 + PC.ae.C40.6 + PC.ae.C42.1 + PC.ae.C42.2 + PC.ae.C42.3 + PC.ae.C42.4 + PC.ae.C42.5 + PC.ae.C44.3 + PC.ae.C44.4 + PC.ae.C44.5 + PC.ae.C44.6) |
| Sum of Lysophosphatidylcholines | total_lysoPC | lysoPC.a.C16.0 + lysoPC.a.C16.1 + lysoPC.a.C17.0 + lysoPC.a.C18.0 + lysoPC.a.C18.1 + lysoPC.a.C18.2 + lysoPC.a.C20.3 + lysoPC.a.C20.4 |
| Sum of Monounsaturated Fatty Acid Lysophosphatidylcholines | mufa_lysoPC | lysoPC.a.C16.1 + lysoPC.a.C18.1 |
| Sum of Polyunsaturated Fatty Acid Lysophosphatidylcholines | pufa_lysoPC | lysoPC.a.C18.2 + lysoPC.a.C20.3 + lysoPC.a.C20.4 |
| Sum of Saturated Fatty Acid Lysophosphatidylcholines | sfa_lysoPC | lysoPC.a.C16.0 + lysoPC.a.C17.0 + lysoPC.a.C18.0 |
|  |  |  |
| Ratio of Monounsaturated Fatty Acid Phosphatidylcholines to Saturated Fatty  Acid Phosphatidylcholines | mufaPC_sfaPC | (PC.aa.C28.1 + PC.aa.C32.1 + PC.aa.C34.1 + PC.aa.C36.1 + PC.aa.C38.1 + PC.aa.C42.1 + PC.ae.C32.1 + PC.ae.C34.1 + PC.ae.C36.1 + PC.ae.C38.1 + PC.ae.C40.1 + PC.ae.C42.1) / (PC.aa.C30.0 + PC.aa.C32.0 + PC.aa.C38.0 + PC.aa.C42.0 + PC.ae.C30.0 + PC.ae.C34.0 + PC.ae.C36.0 + PC.ae.C38.0) |
| Ratio of Acyl-Alkyl-Phosphatidylcholines to Diacyl-Phosphatidylcholines | PCae_PCaa | (PC.ae.C30.0 + PC.ae.C32.1 + PC.ae.C32.2 + PC.ae.C34.0 + PC.ae.C34.1 + PC.ae.C34.2 + PC.ae.C34.3 + PC.ae.C36.0 + PC.ae.C36.1 + PC.ae.C36.2 + PC.ae.C36.3 + PC.ae.C36.4 + PC.ae.C36.5 + PC.ae.C38.0 + PC.ae.C38.1 + PC.ae.C38.2 + PC.ae.C38.3 + PC.ae.C38.4 + PC.ae.C38.5 + PC.ae.C38.6 + PC.ae.C40.1 + PC.ae.C40.2 + PC.ae.C40.3 + PC.ae.C40.4 + PC.ae.C40.5 + PC.ae.C40.6 + PC.ae.C42.1 + PC.ae.C42.2 + PC.ae.C42.3 + PC.ae.C42.4 + PC.ae.C42.5 + PC.ae.C44.3 + PC.ae.C44.4 + PC.ae.C44.5 + PC.ae.C44.6) / (PC.aa.C28.1 + PC.aa.C30.0 + PC.aa.C32.0 + PC.aa.C32.1 + PC.aa.C32.2 + PC.aa.C32.3 + PC.aa.C34.1 + PC.aa.C34.2 + PC.aa.C34.3 + PC.aa.C34.4 + PC.aa.C36.1 + PC.aa.C36.2 + PC.aa.C36.3 + PC.aa.C36.4 + PC.aa.C36.5 + PC.aa.C36.6 + PC.aa.C38.0 + PC.aa.C38.1 + PC.aa.C38.3 + PC.aa.C38.4 + PC.aa.C38.5 + PC.aa.C38.6 + PC.aa.C40.2 + PC.aa.C40.3 + PC.aa.C40.4 + PC.aa.C40.5 + PC.aa.C40.6 + PC.aa.C42.0 + PC.aa.C42.1 + PC.aa.C42.2 + PC.aa.C42.4 + PC.aa.C42.5 + PC.aa.C42.6) |
| Ratio of Polyunsaturated Fatty Acid Phosphatidylcholines to Monounsaturated Fatty Acid Phosphatidylcholines | pufaPC_mufaPC | (PC.aa.C32.2 + PC.aa.C32.3 + PC.aa.C34.2 + PC.aa.C34.3 + PC.aa.C34.4 + PC.aa.C36.2 + PC.aa.C36.3 + PC.aa.C36.4 + PC.aa.C36.5 + PC.aa.C36.6 + PC.aa.C38.3 + PC.aa.C38.4 + PC.aa.C38.5 + PC.aa.C38.6 + PC.aa.C40.2 + PC.aa.C40.3 + PC.aa.C40.4 + PC.aa.C40.5 + PC.aa.C40.6 + PC.aa.C42.2 + PC.aa.C42.4 + PC.aa.C42.5 + PC.aa.C42.6 + PC.ae.C32.2 + PC.ae.C34.2 + PC.ae.C34.3 + PC.ae.C36.2 + PC.ae.C36.3 + PC.ae.C36.4 + PC.ae.C36.5 + PC.ae.C38.2 + PC.ae.C38.3 + PC.ae.C38.4 + PC.ae.C38.5 + PC.ae.C38.6 + PC.ae.C40.2 + PC.ae.C40.3 + PC.ae.C40.4 + PC.ae.C40.5 + PC.ae.C40.6 + PC.ae.C42.1 + PC.ae.C42.2 + PC.ae.C42.3 + PC.ae.C42.4 + PC.ae.C42.5 + PC.ae.C44.3 + PC.ae.C44.4 + PC.ae.C44.5 + PC.ae.C44.6) / (PC.aa.C28.1 + PC.aa.C32.1 + PC.aa.C34.1 + PC.aa.C36.1 + PC.aa.C38.1 + PC.aa.C42.1 + PC.ae.C32.1 + PC.ae.C34.1 + PC.ae.C36.1 + PC.ae.C38.1 + PC.ae.C40.1 + PC.ae.C42.1) |
| Ratio of Polyunsaturated Fatty Acid Phosphatidylcholines to Saturated Fatty  Acid Phosphatidylcholines | pufaPC_sfaPC | (PC.aa.C32.2 + PC.aa.C32.3 + PC.aa.C34.2 + PC.aa.C34.3 + PC.aa.C34.4 + PC.aa.C36.2 + PC.aa.C36.3 + PC.aa.C36.4 + PC.aa.C36.5 + PC.aa.C36.6 + PC.aa.C38.3 + PC.aa.C38.4 + PC.aa.C38.5 + PC.aa.C38.6 + PC.aa.C40.2 + PC.aa.C40.3 + PC.aa.C40.4 + PC.aa.C40.5 + PC.aa.C40.6 + PC.aa.C42.2 + PC.aa.C42.4 + PC.aa.C42.5 + PC.aa.C42.6 + PC.ae.C32.2 + PC.ae.C34.2 + PC.ae.C34.3 + PC.ae.C36.2 + PC.ae.C36.3 + PC.ae.C36.4 + PC.ae.C36.5 + PC.ae.C38.2 + PC.ae.C38.3 + PC.ae.C38.4 + PC.ae.C38.5 + PC.ae.C38.6 + PC.ae.C40.2 + PC.ae.C40.3 + PC.ae.C40.4 + PC.ae.C40.5 + PC.ae.C40.6 + PC.ae.C42.1 + PC.ae.C42.2 + PC.ae.C42.3 + PC.ae.C42.4 + PC.ae.C42.5 + PC.ae.C44.3 + PC.ae.C44.4 + PC.ae.C44.5 + PC.ae.C44.6) / (PC.aa.C30.0 + PC.aa.C32.0 + PC.aa.C38.0 + PC.aa.C42.0 + PC.ae.C30.0 + PC.ae.C34.0 + PC.ae.C36.0 + PC.ae.C38.0) |
| Sum of Monounsaturated Fatty Acid Phosphatidylcholines | total_mufaPCax | PC.aa.C28.1 + PC.aa.C32.1 + PC.aa.C34.1 + PC.aa.C36.1 + PC.aa.C38.1 + PC.aa.C42.1 + PC.ae.C32.1 + PC.ae.C34.1 + PC.ae.C36.1 + PC.ae.C38.1 + PC.ae.C40.1 + PC.ae.C42.1 |
| Sum of Phosphatidylcholines | total_PCax | PC.aa.C28.1 + PC.aa.C30.0 + PC.aa.C32.0 + PC.aa.C32.1 + PC.aa.C32.2 + PC.aa.C32.3 + PC.aa.C34.1 + PC.aa.C34.2 + PC.aa.C34.3 + PC.aa.C34.4 + PC.aa.C36.1 + PC.aa.C36.2 + PC.aa.C36.3 + PC.aa.C36.4 + PC.aa.C36.5 + PC.aa.C36.6 + PC.aa.C38.0 + PC.aa.C38.1 + PC.aa.C38.3 + PC.aa.C38.4 + PC.aa.C38.5 + PC.aa.C38.6 + PC.aa.C40.2 + PC.aa.C40.3 + PC.aa.C40.4 + PC.aa.C40.5 + PC.aa.C40.6 + PC.aa.C42.0 + PC.aa.C42.1 + PC.aa.C42.2 + PC.aa.C42.4 + PC.aa.C42.5 + PC.aa.C42.6 + PC.ae.C30.0 + PC.ae.C32.1 + PC.ae.C32.2 + PC.ae.C34.0 + PC.ae.C34.1 + PC.ae.C34.2 + PC.ae.C34.3 + PC.ae.C36.0 + PC.ae.C36.1 + PC.ae.C36.2 + PC.ae.C36.3 + PC.ae.C36.4 + PC.ae.C36.5 + PC.ae.C38.0 + PC.ae.C38.1 + PC.ae.C38.2 + PC.ae.C38.3 + PC.ae.C38.4 + PC.ae.C38.5 + PC.ae.C38.6 + PC.ae.C40.1 + PC.ae.C40.2 + PC.ae.C40.3 + PC.ae.C40.4 + PC.ae.C40.5 + PC.ae.C40.6 + PC.ae.C42.1 + PC.ae.C42.2 + PC.ae.C42.3 + PC.ae.C42.4 + PC.ae.C42.5 + PC.ae.C44.3 + PC.ae.C44.4 + PC.ae.C44.5 + PC.ae.C44.6 |
| Sum of Diacyl-Phosphatidylcholines | total_PCaa | PC.aa.C28.1 + PC.aa.C30.0 + PC.aa.C32.0 + PC.aa.C32.1 + PC.aa.C32.2 + PC.aa.C32.3 + PC.aa.C34.1 + PC.aa.C34.2 + PC.aa.C34.3 + PC.aa.C34.4 + PC.aa.C36.1 + PC.aa.C36.2 + PC.aa.C36.3 + PC.aa.C36.4 + PC.aa.C36.5 + PC.aa.C36.6 + PC.aa.C38.0 + PC.aa.C38.1 + PC.aa.C38.3 + PC.aa.C38.4 + PC.aa.C38.5 + PC.aa.C38.6 + PC.aa.C40.2 + PC.aa.C40.3 + PC.aa.C40.4 + PC.aa.C40.5 + PC.aa.C40.6 + PC.aa.C42.0 + PC.aa.C42.1 + PC.aa.C42.2 + PC.aa.C42.4 + PC.aa.C42.5 + PC.aa.C42.6 |
| Sum of Acyl-Alkyl-Phosphatidylcholines | total_PCae | PC.ae.C30.0 + PC.ae.C32.1 + PC.ae.C32.2 + PC.ae.C34.0 + PC.ae.C34.1 + PC.ae.C34.2 + PC.ae.C34.3 + PC.ae.C36.0 + PC.ae.C36.1 + PC.ae.C36.2 + PC.ae.C36.3 + PC.ae.C36.4 + PC.ae.C36.5 + PC.ae.C38.0 + PC.ae.C38.1 + PC.ae.C38.2 + PC.ae.C38.3 + PC.ae.C38.4 + PC.ae.C38.5 + PC.ae.C38.6 + PC.ae.C40.1 + PC.ae.C40.2 + PC.ae.C40.3 + PC.ae.C40.4 + PC.ae.C40.5 + PC.ae.C40.6 + PC.ae.C42.1 + PC.ae.C42.2 + PC.ae.C42.3 + PC.ae.C42.4 + PC.ae.C42.5 + PC.ae.C44.3 + PC.ae.C44.4 + PC.ae.C44.5 + PC.ae.C44.6 |
| Sum of Polyunsaturated Fatty Acid Phosphatidylcholines | total_pufaPCax | PC.aa.C32.2 + PC.aa.C32.3 + PC.aa.C34.2 + PC.aa.C34.3 + PC.aa.C34.4 + PC.aa.C36.2 + PC.aa.C36.3 + PC.aa.C36.4 + PC.aa.C36.5 + PC.aa.C36.6 + PC.aa.C38.3 + PC.aa.C38.4 + PC.aa.C38.5 + PC.aa.C38.6 + PC.aa.C40.2 + PC.aa.C40.3 + PC.aa.C40.4 + PC.aa.C40.5 + PC.aa.C40.6 + PC.aa.C42.2 + PC.aa.C42.4 + PC.aa.C42.5 + PC.aa.C42.6 + PC.ae.C32.2 + PC.ae.C34.2 + PC.ae.C34.3 + PC.ae.C36.2 + PC.ae.C36.3 + PC.ae.C36.4 + PC.ae.C36.5 + PC.ae.C38.2 + PC.ae.C38.3 + PC.ae.C38.4 + PC.ae.C38.5 + PC.ae.C38.6 + PC.ae.C40.2 + PC.ae.C40.3 + PC.ae.C40.4 + PC.ae.C40.5 + PC.ae.C40.6 + PC.ae.C42.1 + PC.ae.C42.2 + PC.ae.C42.3 + PC.ae.C42.4 + PC.ae.C42.5 + PC.ae.C44.3 + PC.ae.C44.4 + PC.ae.C44.5 + PC.ae.C44.6 |
| Sum of Saturated Fatty Acid Phosphatidylcholines | total_sfaPCax | PC.aa.C30.0 + PC.aa.C32.0 + PC.aa.C38.0 + PC.aa.C42.0 + PC.ae.C30.0 + PC.ae.C34.0 + PC.ae.C36.0 + PC.ae.C38.0 |
|  |  |  |
| Ratio of Hydroxylated Sphingomyelins to  Non-Hydroxylated Sphingomyelins | SM.OH_SMnonOH | (SM..OH..C14.1 + SM..OH..C16.1 + SM..OH..C22.1 + SM..OH..C22.2 + SM..OH..C24.1) / (SM.C16.0 + SM.C16.1 + SM.C18.0 + SM.C18.1 + SM.C20.2 + SM.C24.0 + SM.C24.1) |
| Ratio of Sphingomyelins to Phosphatidylcholines | SM_PC | (SM..OH..C14.1 + SM..OH..C16.1 + SM..OH..C22.1 + SM..OH..C22.2 + SM..OH..C24.1 + SM.C16.0 + SM.C16.1 + SM.C18.0 + SM.C18.1 + SM.C20.2 + SM.C24.0 + SM.C24.1) / (PC.aa.C28.1 + PC.aa.C30.0 + PC.aa.C32.0 + PC.aa.C32.1 + PC.aa.C32.2 + PC.aa.C32.3 + PC.aa.C34.1 + PC.aa.C34.2 + PC.aa.C34.3 + PC.aa.C34.4 + PC.aa.C36.1 + PC.aa.C36.2 + PC.aa.C36.3 + PC.aa.C36.4 + PC.aa.C36.5 + PC.aa.C36.6 + PC.aa.C38.0 + PC.aa.C38.1 + PC.aa.C38.3 + PC.aa.C38.4 + PC.aa.C38.5 + PC.aa.C38.6 + PC.aa.C40.2 + PC.aa.C40.3 + PC.aa.C40.4 + PC.aa.C40.5 + PC.aa.C40.6 + PC.aa.C42.0 + PC.aa.C42.1 + PC.aa.C42.2 + PC.aa.C42.4 + PC.aa.C42.5 + PC.aa.C42.6 + PC.ae.C30.0 + PC.ae.C32.1 + PC.ae.C32.2 + PC.ae.C34.0 + PC.ae.C34.1 + PC.ae.C34.2 + PC.ae.C34.3 + PC.ae.C36.0 + PC.ae.C36.1 + PC.ae.C36.2 + PC.ae.C36.3 + PC.ae.C36.4 + PC.ae.C36.5 + PC.ae.C38.0 + PC.ae.C38.1 + PC.ae.C38.2 + PC.ae.C38.3 + PC.ae.C38.4 + PC.ae.C38.5 + PC.ae.C38.6 + PC.ae.C40.1 + PC.ae.C40.2 + PC.ae.C40.3 + PC.ae.C40.4 + PC.ae.C40.5 + PC.ae.C40.6 + PC.ae.C42.1 + PC.ae.C42.2 + PC.ae.C42.3 + PC.ae.C42.4 + PC.ae.C42.5 + PC.ae.C44.3 + PC.ae.C44.4 + PC.ae.C44.5 + PC.ae.C44.6) |
| Sum of Non-Hydroxylated Sphingomyelins | total_SM_nonOH | SM.C16.0 + SM.C16.1 + SM.C18.0 + SM.C18.1 + SM.C20.2 + SM.C24.0 + SM.C24.1 |
| Sum of Hydroxylated Sphingomyelins | total_SM.OH | SM..OH..C14.1 + SM..OH..C16.1 + SM..OH..C22.1 + SM..OH..C22.2 + SM..OH..C24.1 |
| Sum of Sphingomyelins | total_SM | SM..OH..C14.1 + SM..OH..C16.1 + SM..OH..C22.1 + SM..OH..C22.2 + SM..OH..C24.1 + SM.C16.0 + SM.C16.1 + SM.C18.0 + SM.C18.1 + SM.C20.2 + SM.C24.0 + SM.C24.1 |

**Supplementary Table 2:** Demographic and clinical characteristics of the sample, stratified by sex

|  | **Overall** | **Men** | **Women** |
| --- | --- | --- | --- |
| **n** | **376** | **217** | **159** |
| Age, years | 56.4 (9.2) | 56.5 (9.3) | 56.3 (9.1) |
| Weight, kg | 82.9 (16.7) | 89.9 (14.5) | 73.3 (14.5) |
| Height, cm | 171.8 (9.8) | 177.9 (6.8) | 163.4 (6.6) |
| BMI, kg/m^2^ | 28.0 (4.9) | 28.4 (4.5) | 27.5 (5.4) |
| Waist circumference, cm | 98.4 (14.4) | 103.5 (12.6) | 91.6 (13.8) |
| Post menopausale | 106 (66.7) | <NA> | 106 (66.7) |
| Alcohol consumption, g/day (median [Q1, Q3]) | 8.6 [0.2, 26.2] | 20.0 [3.7, 40.0] | 2.9 [0, 12.3] |
| Smoking behaviour |  |  |  |
| never | 137 (36.4) | 71 (32.7) | 66 (41.5) |
| former | 164 (43.6) | 105 (48.4) | 59 (37.1) |
| current | 75 (19.9) | 41 (18.9) | 34 (21.4) |
| Systolic blood pressure, mmHg | 120.6 (16.9) | 126.2 (16.4) | 112.9 (14.3) |
| Diastolic blood pressure, mmHg | 75.3 (10.0) | 77.7 (10.4) | 72.1 (8.5) |
| Hypertension | 129 (34.3) | 83 (38.2) | 46 (28.9) |
| Antihypertensive medication | 97 (25.8) | 53 (24.4) | 44 (27.7) |
| Total cholesterol, mg/dL | 217.6 (36.6) | 216.8 (37.9) | 218.8 (34.8) |
| Triglycerides, mg/dL | 131.7 (86.7) | 153.6 (101.9) | 101.9 (45.9) |
| LDL, mg/dL | 139.4 (33.1) | 141.6 (33.8) | 136.3 (32.0) |
| Lipid lowering medication | 41 (10.9) | 24 (11.1) | 17 (10.7) |
| Glycemia |  |  |  |
| normoglycemic | 232 (61.7) | 118 (54.4) | 114 (71.7) |
| Prediabetes | 91 (24.2) | 59 (27.2) | 32 (20.1) |
| T2 Diabetes | 53 (14.1) | 40 (18.4) | 13 (8.2) |
| HbA1c, % | 5.6 (0.7) | 5.6 (0.8) | 5.5 (0.5) |
| Fasting glucose, mg/dL | 104.1 (22.9) | 108.1 (25.5) | 98.7 (17.4) |
| Fasting insulin, mg/dL | 11.2 (7.7) | 12.4 (8.7) | 9.7 (5.7) |
| Serum uric acid, mg/dL | 5.6 (1.5) | 6.4 (1.3) | 4.6 (1.1) |
| hsCRP, mg/L (median [Q1, Q3]) | 1.18 [0.61, 2.47] | 1.12 [0.60, 2.32] | 1.34 [0.69, 2.77] |
|  |  |  |  |
| **Hepatic phenotypes** |  |  |  |
| Hepatic fat content, % | 8.9 (8.1) | 10.8 (8.8) | 6.2 (6.2) |
| Hepatic fat content, %, median [IQR] | 5.7 [9.1] | 7.4 [12.1] | 3.8 [5.8] |
| Steatosis | 191 (50.8) | 139 (64.1) | 52 (32.7) |
| Hepatic iron content, 1/s | 40.6 (4.7) | 41.7 (4.9) | 39.2 (4.1) |
| Iron overload | 164 (43.6) | 120 (55.3) | 44 (27.7) |
| ALT (GPT), μkat/l | 0.52 (0.29) | 0.59 (0.29) | 0.41 (0.25) |
| AST (GOT), μkat/l | 0.42 (0.22) | 0.46 (0.23) | 0.37 (0.18) |
| GGT, μkat/l | 0.66 (0.67) | 0.78 (0.73) | 0.49 (0.54) |
| Fatty liver index, continuous | 54.2 (31.3) | 63.8 (27.2) | 41.1 (31.9) |
| <30 | 109 (29.0) | 33 (15.2) | 76 (47.8) |
| ≥30 and <60 | 86 (22.9) | 57 (26.3) | 29 (18.2) |
| ≥60 | 181 (48.1) | 127 (58.5) | 54 (34.0) |

Presented are mean (SD) for continuous data or n (%) for categorical data if not stated differently. Steatosis was defined as hepatic fat content ≥ 5.56%. Iron overload was defined as hepatic iron content ≥ 41 1/s.

**Supplementary Table 3:** Results from pathway analysis of individuals with steatosis compared to individuals without steatosis

|  | Hits of Total Compounds | Raw p-value | -log10(p) | Impact |
| --- | --- | --- | --- | --- |
| Glycerophospholipid metabolism | 2 / 36 | 6.583e-13 | 12.182 | 0.11 |
| Alanine, aspartate and glutamate metabolism | 3 / 28 | 4.1681e-12 | 11.38 | 0.42 |
| D-Glutamine and D-glutamate metabolism | 1 / 6 | 2.2306e-11 | 10.652 | 0.5 |
| Arginine and proline metabolism | 1 / 38 | 2.2306e-11 | 10.652 | 0.09 |
| Glutathione metabolism | 1 / 28 | 2.2306e-11 | 10.652 | 0.02 |
| Glyoxylate and dicarboxylate metabolism | 1 / 32 | 2.2306e-11 | 10.652 | 0 |
| Butanoate metabolism | 1 / 15 | 2.2306e-11 | 10.652 | 0 |
| Porphyrin and chlorophyll metabolism | 1 / 30 | 2.2306e-11 | 10.652 | 0 |
| Nitrogen metabolism | 1 / 6 | 2.2306e-11 | 10.652 | 0 |
| Aminoacyl-tRNA biosynthesis | 6 / 48 | 4.0952e-10 | 9.3877 | 0 |
| Arginine biosynthesis | 2 / 14 | 9.1821e-09 | 8.0371 | 0.12 |
| Histidine metabolism | 2 / 16 | 9.1821e-09 | 8.0371 | 0 |
| Selenocompound metabolism | 1 / 20 | 1.5976e-08 | 7.7965 | 0 |
| Lysine degradation | 1 / 25 | 1.7328e-07 | 6.7613 | 0.14 |
| Valine, leucine and isoleucine degradation | 2 / 40 | 5.9381e-05 | 4.2263 | 0 |
| Valine, leucine and isoleucine biosynthesis | 2 / 8 | 5.9381e-05 | 4.2263 | 0 |
| Phenylalanine, tyrosine and tryptophan biosynthesis | 1 / 4 | 0.0032891 | 2.4829 | 0.5 |
| Phenylalanine metabolism | 1 / 10 | 0.0032891 | 2.4829 | 0.36 |
| Arachidonic acid metabolism | 1 / 36 | 0.0044302 | 2.3536 | 0 |
| Linoleic acid metabolism | 1 / 5 | 0.0044302 | 2.3536 | 0 |
| alpha-Linolenic acid metabolism | 1 / 13 | 0.0044302 | 2.3536 | 0 |
| Sphingolipid metabolism | 1 / 21 | 0.034765 | 1.4589 | 0 |
| beta-Alanine metabolism | 1 / 21 | 0.37195 | 0.42951 | 0 |
| Nicotinate and nicotinamide metabolism | 1 / 15 | 0.37195 | 0.42951 | 0 |
| Pantothenate and CoA biosynthesis | 1 / 19 | 0.37195 | 0.42951 | 0 |

**Supplementary Table 4:** Results from pathway analysis of individuals with iron overload compared to individuals without iron overload

|  | Hits of Total Compounds | Raw p-value | -log10(p) | Impact |
| --- | --- | --- | --- | --- |
| Arachidonic acid metabolism | 1 / 36 | 1.2966e-05 | 4.8872 | 0 |
| Linoleic acid metabolism | 1 / 5 | 1.2966e-05 | 4.8872 | 0 |
| alpha-Linolenic acid metabolism | 1 / 13 | 1.2966e-05 | 4.8872 | 0 |
| Glycerophospholipid metabolism | 2 / 36 | 5.3843e-05 | 4.2689 | 0.11 |
| Lysine degradation | 1 / 25 | 0.00091127 | 3.0404 | 0.14 |
| Valine, leucine and isoleucine degradation | 2 / 40 | 0.0074542 | 2.1276 | 0 |
| Valine, leucine and isoleucine biosynthesis | 2 / 8 | 0.0074542 | 2.1276 | 0 |
| Aminoacyl-tRNA biosynthesis | 6 / 48 | 0.020573 | 1.6867 | 0 |
| Sphingolipid metabolism | 1 / 21 | 0.075959 | 1.1194 | 0 |
| Phenylalanine, tyrosine and tryptophan biosynthesis | 1 / 4 | 0.076152 | 1.1183 | 0.5 |
| Phenylalanine metabolism | 1 / 10 | 0.076152 | 1.1183 | 0.36 |
| Selenocompound metabolism | 1 / 20 | 0.18562 | 0.73138 | 0 |
| Alanine, aspartate and glutamate metabolism | 3 / 28 | 0.3927 | 0.40594 | 0.42 |
| beta-Alanine metabolism | 1 / 21 | 0.45234 | 0.34454 | 0 |
| Nicotinate and nicotinamide metabolism | 1 / 15 | 0.45234 | 0.34454 | 0 |
| Pantothenate and CoA biosynthesis | 1 / 19 | 0.45234 | 0.34454 | 0 |
| D-Glutamine and D-glutamate metabolism | 1 / 6 | 0.45313 | 0.34378 | 0.5 |
| Arginine and proline metabolism | 1 / 38 | 0.45313 | 0.34378 | 0.09 |
| Glutathione metabolism | 1 / 28 | 0.45313 | 0.34378 | 0.02 |
| Glyoxylate and dicarboxylate metabolism | 1 / 32 | 0.45313 | 0.34378 | 0 |
| Butanoate metabolism | 1 / 15 | 0.45313 | 0.34378 | 0 |
| Porphyrin and chlorophyll metabolism | 1 / 30 | 0.45313 | 0.34378 | 0 |
| Nitrogen metabolism | 1 / 6 | 0.45313 | 0.34378 | 0 |
| Arginine biosynthesis | 2 / 14 | 0.55579 | 0.25509 | 0.12 |
| Histidine metabolism | 2 / 16 | 0.55579 | 0.25509 | 0 |

**Supplementary Table 5:** Areas under the Curve (AUC) from logistic regression models predicting hepatic steatosis and hepatic iron overload.

| **Outcome: Group** | **Predictor** | **AUC** | **p-value** |
| --- | --- | --- | --- |
| Hepatic steatosis: All | *PNPLA3* SNP | 0.625 | 0.0048 |
|  | *HFE* SNP | 0.892 | <0.001 |
|  | Hepatic iron content | 0.731 | Reference |
| Hepatic steatosis: Men | *PNPLA3* SNP | 0.62 | 0.2641 |
|  | *HFE* SNP | 0.89 | <0.001 |
|  | Hepatic iron content | 0.672 | Reference |
| Hepatic steatosis: Women | *PNPLA3* SNP | 0.712 | 0.5664 |
|  | *HFE* SNP | 0.902 | 0.0174 |
|  | Hepatic iron content | 0.747 | Reference |
| Hepatic iron overload: All | *PNPLA3* SNP | 0.676 | 0.2611 |
|  | *HFE* SNP | 0.875 | <0.001 |
|  | Hepatic fat content | 0.726 | 1 |
| Hepatic iron overload: Men | *PNPLA3* SNP | 1 | <0.001 |
|  | *HFE* SNP | 0.889 | <0.001 |
|  | Hepatic fat content | 0.674 | Reference |
| Hepatic iron overload: Women | *PNPLA3* SNP | 0.667 | 0.8511 |
|  | *HFE* SNP | 0.84 | 0.0421 |
|  | Hepatic fat content | 0.69 | Reference |

**Supplementary Figure 1**: Participant flowchart


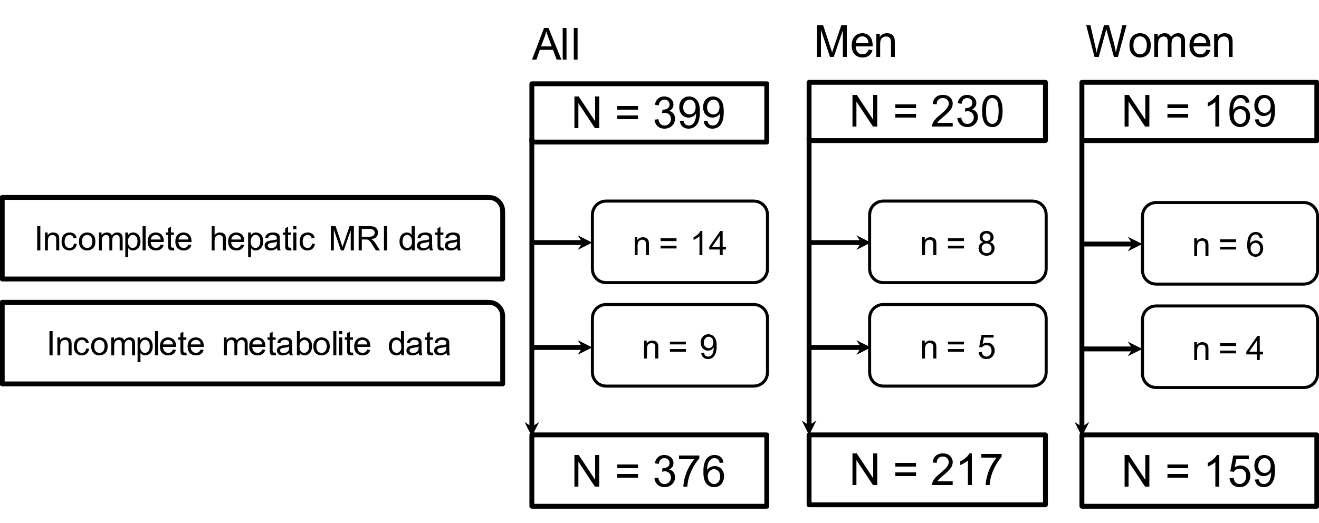


**Supplementary Figure 2:** Correlation of log-transformed hepatic fat content and hepatic iron content. Correlation coefficients are estimated using Spearman correlation and p-values are extracted from unadjusted linear regression models.


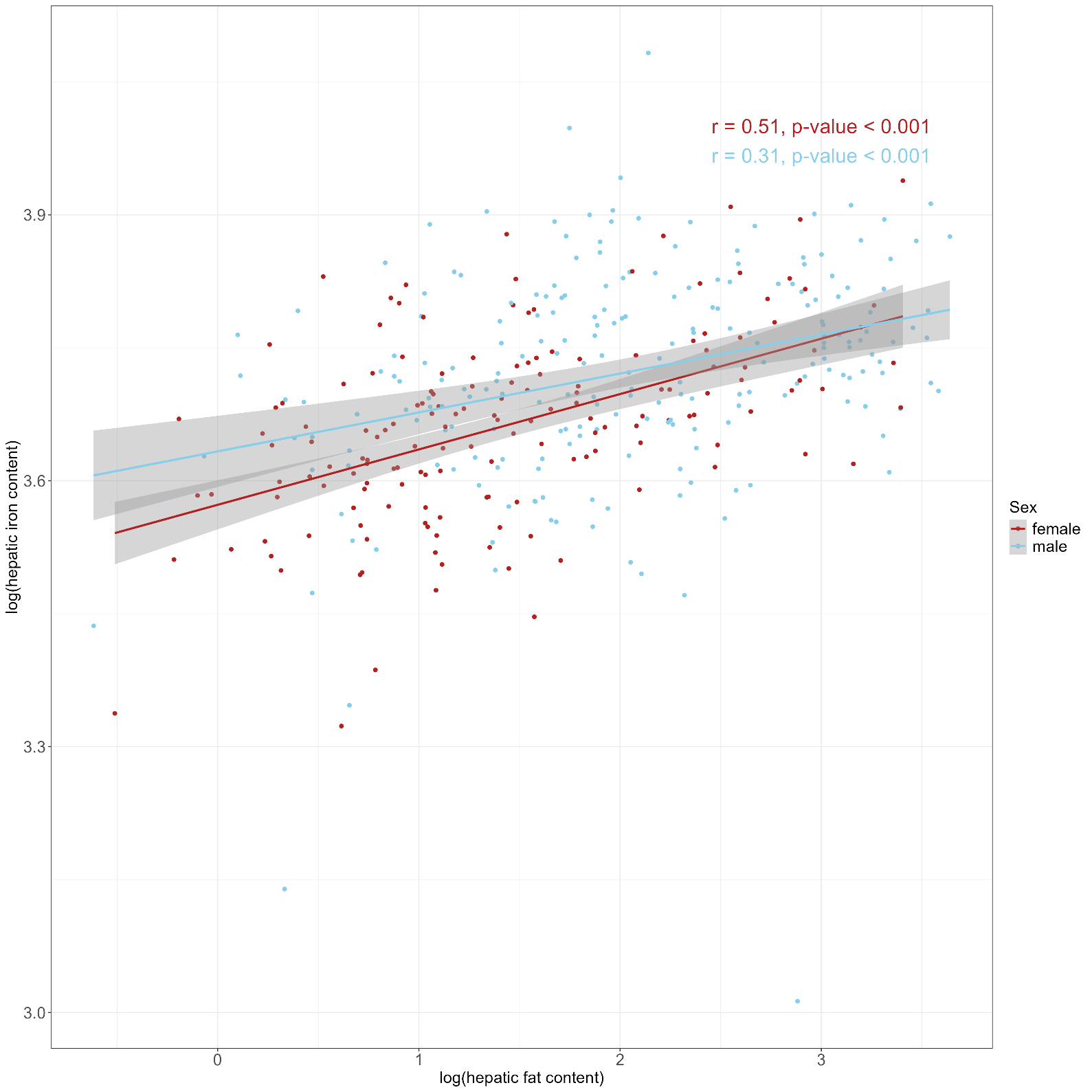


**Supplementary Figure 3:** PCA plots of metabolite data with colors indicating the plate.


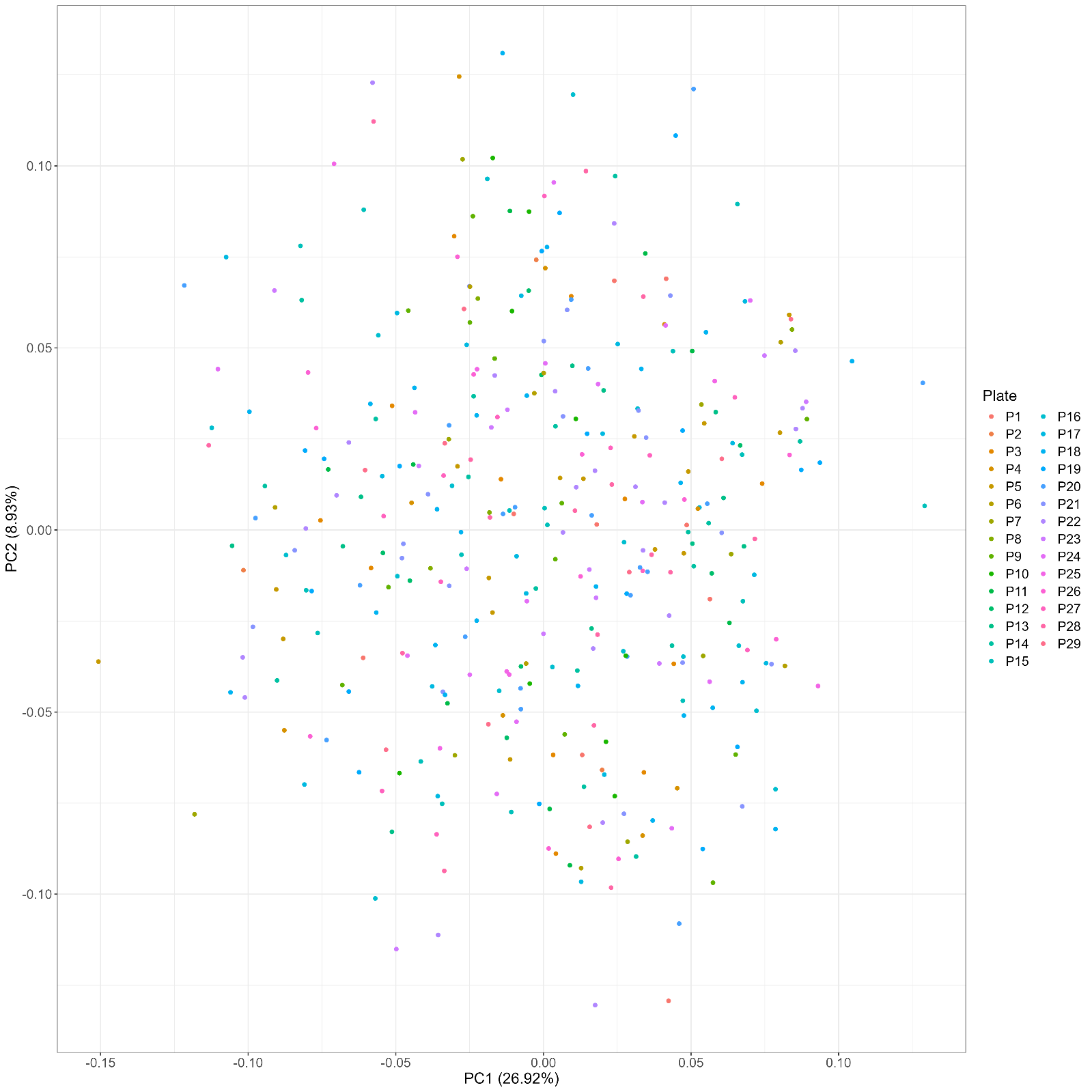


**Supplementary Figure 4:** Forest plot of all significant associations between metabolites and hepatic phenotypes. The x-axis shows the effect estimate with 95% confidence interval and the y-axis the metabolites. The colors of dots and column names represent the hepatic phenotype. Significant associations are shown in bold and non-significant associations in transparent.


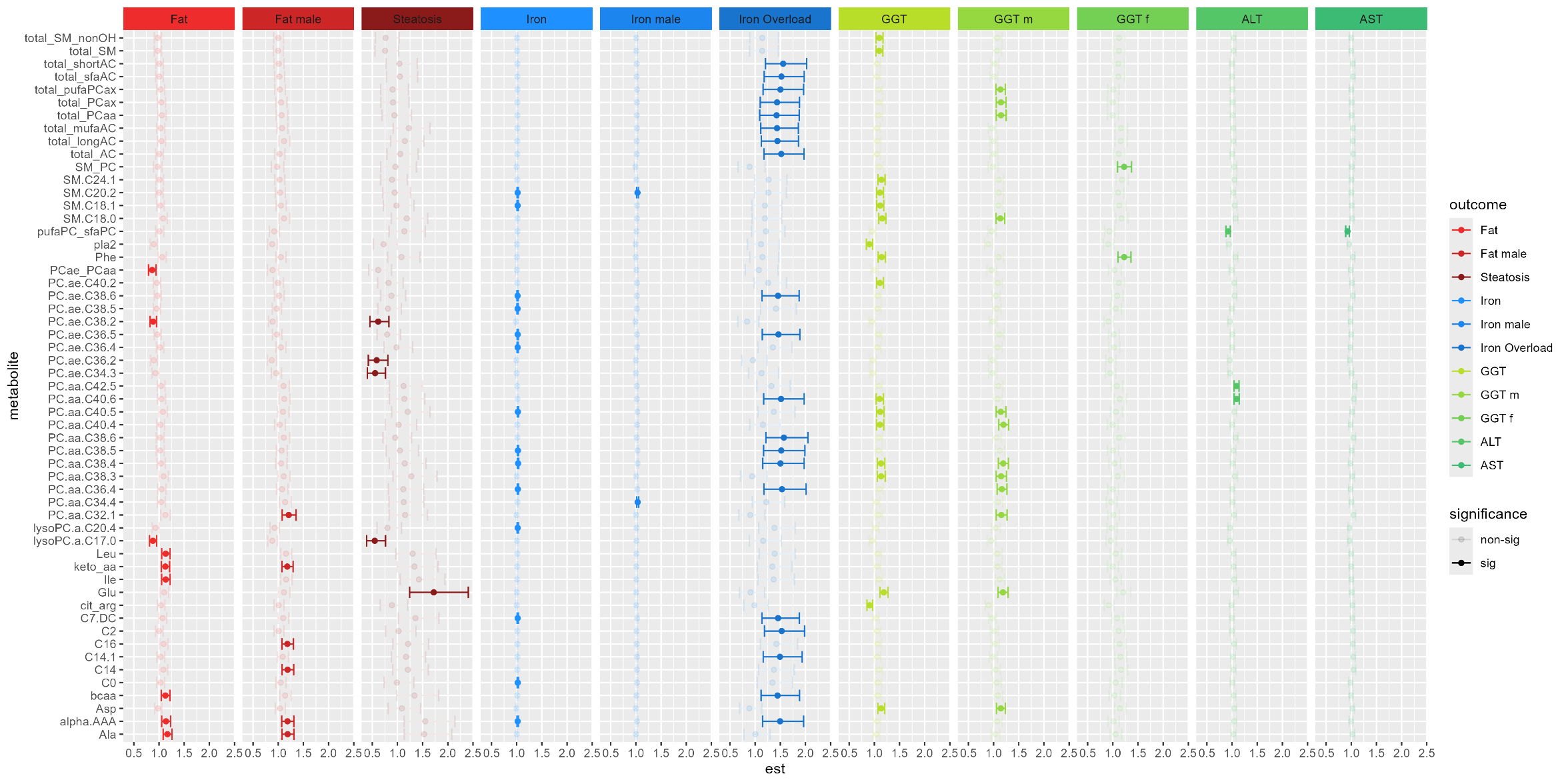

Supplement: Supplementary file 3 — Additional file 3. [file 10020_2025_1309_MOESM3_ESM.docx]
